# Polysulfide metabolizing enzymes influence SqrR-mediated sulfide-induced transcription by impacting intracellular polysulfide dynamics

**DOI:** 10.1093/pnasnexus/pgad048

**Published:** 2023-02-10

**Authors:** Takayuki Shimizu, Tomoaki Ida, Giuliano T Antelo, Yuta Ihara, Joseph N Fakhoury, Shinji Masuda, David P Giedroc, Takaaki Akaike, Daiana A Capdevila, Tatsuru Masuda

**Affiliations:** Graduate School of Arts and Sciences, The University of Tokyo, 3-8-1 Komaba, Meguro-ku, Tokyo 153-8902, Japan; Department of 8 Environmental Medicine and Molecular Toxicology, Tohoku University Graduate School of Medicine, 2-1 Seiryo-machi, Aoba-ku, Sendai 980-8575, Japan; Department of Chemistry, Indiana University, 800 E. Kirkwood Dr, Bloomington, IN 47405-7102, USA; Department of Molecular and Cellular Biochemistry, Indiana University, 212 S. Hawthorne Dr, Bloomington, IN 47405, USA; Fundación Instituto Leloir, Instituto de Investigaciones Bioquímicas de Buenos Aires (IIBBA-CONICET), Av. Patricias Argentinas 435, Buenos Aires C1405BWE, Argentina; Department of Life Science and Technology, Tokyo Institute of Technology, 4259 Nagatsuta-cho, Midori-ku, Yokohama 226-8501, Japan; Department of Chemistry, Indiana University, 800 E. Kirkwood Dr, Bloomington, IN 47405-7102, USA; Department of Life Science and Technology, Tokyo Institute of Technology, 4259 Nagatsuta-cho, Midori-ku, Yokohama 226-8501, Japan; Department of Chemistry, Indiana University, 800 E. Kirkwood Dr, Bloomington, IN 47405-7102, USA; Department of Molecular and Cellular Biochemistry, Indiana University, 212 S. Hawthorne Dr, Bloomington, IN 47405, USA; Department of 8 Environmental Medicine and Molecular Toxicology, Tohoku University Graduate School of Medicine, 2-1 Seiryo-machi, Aoba-ku, Sendai 980-8575, Japan; Department of Chemistry, Indiana University, 800 E. Kirkwood Dr, Bloomington, IN 47405-7102, USA; Department of Molecular and Cellular Biochemistry, Indiana University, 212 S. Hawthorne Dr, Bloomington, IN 47405, USA; Fundación Instituto Leloir, Instituto de Investigaciones Bioquímicas de Buenos Aires (IIBBA-CONICET), Av. Patricias Argentinas 435, Buenos Aires C1405BWE, Argentina; Graduate School of Arts and Sciences, The University of Tokyo, 3-8-1 Komaba, Meguro-ku, Tokyo 153-8902, Japan

**Keywords:** polysulfide, sulfur metabolism, signal transduction, proteobacteria

## Abstract

Sulfide plays essential roles in controlling various physiological activities in almost all organisms. Although recent evidence has demonstrated that sulfide is endogenously generated and metabolized into polysulfides inside the cells, the relationship between polysulfide metabolism and polysulfide-sensing mechanisms is not well understood. To better define this interplay between polysulfide metabolism and sensing in cells, we investigated the role of polysulfide-metabolizing enzymes such as sulfide:quinone oxidoreductase (SQR) on the temporal dynamics of cellular polysulfide speciation and on the transcriptional regulation by the persulfide-responsive transcription factor SqrR in *Rhodobacter capsulatus*. We show that disruption of the *sqr* gene resulted in the loss of SqrR repression by exogenous sulfide at longer culture times, which impacts the speciation of intracellular polysulfides of Δ*sqr* vs. wild-type strains. Both the attenuated response of SqrR and the change in polysulfide dynamics of the Δ*sqr* strain is fully reversed by the addition to cells of cystine-derived polysulfides, but not by glutathione disulfide (GSSG)-derived polysulfides. Furthermore, cysteine persulfide (CysSSH) yields a higher rate of oxidation of SqrR relative to glutathione persulfide (GSSH), which leads to DNA dissociation *in vitro*. The oxidation of SqrR was confirmed by a mass spectrometry-based kinetic profiling strategy that showed distinct polysulfide-crosslinked products obtained with CysSSH vs. GSSH. Taken together, these results establish a novel association between the metabolism of polysulfides and the mechanisms for polysulfide sensing inside the cells.

**Significance Statement**

Polysulfide sensing and signaling is operative in both prokaryotes and eukaryotes. Although polysulfide metabolism and sensing mechanisms have been investigated in various organisms, how endogenous polysulfide production impacts polysulfide-induced signal transduction is largely unexplored. Here, we show how polysulfide-metabolizing enzymes influence SqrR-mediated polysulfide-induced transcription. These findings provide new insights into persulfide biology and redox physiology. As alphaproteobacteria retain some physiological characteristics of mitochondria, the characterization of SqrR-mediated polysulfide-induced transcription provides new insights into plausible mechanisms of polysulfide homeostasis in diverse organisms.

## Introduction

Hydrogen sulfide (H_2_S) shaped the evolution of early life forms as it is thought to have provided the basic redox chemistry needed by organisms in the pre-oxygenic world (1). Consistently, recent studies reveal an important role(s) of H_2_S in the physiology of nearly all extant organisms (2–5). Many extant bacteria utilize H_2_S as an energy source or electron donor, and it has been reported that sulfide increases bacterial resistance to antibiotics in *Escherichia coli* (2). Moreover, polysulfides derived from hydrogen sulfide modulate various physiological functions, potentially as signaling molecules, although these mechanisms remain poorly understood (6–9). In eukaryotes, sulfide has been historically described by its cytotoxic effect in the inhibition of mitochondrial respiration via coordination to the heme iron in the terminal oxidase (complex IV) (10). A more recent perspective states that low concentrations of sulfide promote oxidative phosphorylation in mitochondria via reduction of ferric iron of heme (11, 12). Moreover, sulfide-dependent respiration by SQR has also been reported in mammalian cells (11, 13). Thus, sulfide possesses both cytotoxic and beneficial effects; therefore, organisms must strictly control the intracellular levels of sulfide and reactive polysulfides to harness their beneficial effects while avoiding their toxicity.

Almost all organisms can generate sulfide from cysteine (Cys) metabolism and/or inorganic sulfur compounds (14, 15) and enzymatically generated polysulfides are ubiquitous in biology. Here, a low molecular weight (LMW) thiol acceptor, e.g. Cys or glutathione, can be oxidized to create a thiol persulfide, RSSH, also known as a hydropersulfide (16–18). Metabolic processes of polysulfide synthesis and degradation have been well studied in mammals, and several enzymes that oxidize sulfide or catalyze the conversion of an LMW thiol to a polysulfide have been characterized. Although SQR is a well-established polysulfide producer in both mammals and bacteria (17–19), the study of other polysulfide-metabolizing enzymes has been restricted to mammals. Here, two transsulfuration enzymes, cystathionine β-synthase (CBS) and cystathionine γ-lyase (CSE), catalyze endogenous CysSSH formation from cystine (6, 7). *L*-cysteine aminotransferase (CAT) and mercaptopyruvate sulfurtransferase (MST) can also generate a CysSSH in the catalytic site of MST from *L*-cysteine via conversion to 3-mercaptopyruvate (20). Further, cysteinyl-tRNA synthetase (CARS) can produce CysSSH from Cys, and the heterozygous mutation of CARS in mice showed ∼50% reduction of CysSSH compared to wild-type mice, suggesting that this is an important pathway of CysSSH formation in mammals (21). Other polysulfides, such as GSSH and inorganic dihydropolysulfide species, are produced via persulfide scrambling (6, 21) and glutathione reductase reduces glutathione trisulfide (GSSSG) to generate GSSH (22). Thus, this metabolic network controls the intracellular polysulfide/persulfide speciation, defined as the concentration of each individual type of polysulfide, which together constitute the total concentration of sulfane sulfur inside the cell.

The sulfuration (or sulfhydration) of various electrophilic species and sulfenylated thiol residues in proteins by H_2_S/HS^–^ is also well established, and the formation of these species is thought to drive polysulfide signal transduction (6–9). In mammals, for example, a small but significant fraction of the proteome is persulfidated (23–26). The same is true for several bacteria, which may provide the organism a readily supply of bioavailable sulfur (27). A number of persulfidated proteins obtain their sulfur atom via transsulfidation (28) and it is known that sulfurtransferases containing a rhodanese homology domain contribute to the transsulfidation of proteins in archaea, bacteria, and eukaryotes (29). Thus, polysulfide-mediated signaling likely impacts various physiological processes throughout the different domains of life. However, the molecular mechanisms of polysulfide signaling and its interplay with the metabolism of these species is not yet fully understood.

Per- and polysulfide sensor proteins have been identified and characterized in several bacteria (30–34). These are transcriptional regulators that modulate the expression of genes encoding enzymes such as SQR by inducing transcriptional de-repression or activation via the formation of reversible per- and polysulfide adducts upon Cys persulfidation. This appears to be a conserved feature between the different polysulfide-sensors in spite of their distinct structures (27, 35). Recently, our laboratories identified the novel polysulfide-responsive transcription factor SqrR as one of these bacterial polysulfide sensors from the alphaproteobacterium *Rhodobacter capsulatus* and revealed its molecular mechanism (31, 35). SqrR represses a significant portion (45%) of the sulfide-responsive genes in the absence of exogenous sulfide. SqrR forms an intramolecular tetrasulfide bond between two conserved Cys residues when exposed to GSSH, reducing its DNA-binding activity *in vitro* (35). An MS-based kinetic profiling experiment characterized this persulfidation process in detail (35, 36), demonstrating that SqrR is specifically oxidized by persulfides and not peroxides or other ROS, as the incorporation of sulfane sulfur atoms by organic persulfides yields a tetrasulfide, which is structurally less “frustrated” relative to the disulfide state that would be formed by ROS (35). Biologically, this allows the organism to avoid crosstalk between the stresses caused by polysulfides and other oxidants, a crucial aspect of the chemistry of these sensors, as physiological concentrations of polysulfides protects against oxidative stress caused by ROS (27). Therefore, SqrR-regulated polysulfide-mediated transcriptional de-repression serves as a model system to investigate (poly)sulfide signaling in cells. In addition, alphaproteobacteria are evolutionary much closer to the ancestral bacteria of mitochondria, because the mitochondrial ancestor may have evolved from a proteobacterial lineage that branched off before the divergence of alphaproteobacteria (37). In fact, *R. capsulatus* encodes at least one persulfide dioxygenase (PDO), a rhodanese and a sulfite oxidase, as well as an SQR, that are core components of the mitochondrial pathway of sulfide oxidation to sulfate of eukaryotic cells (38). Thus, characterization of SqrR-mediated polysulfide signaling in *R. capsulatus* may serve as a model for a better understanding of the polysulfide signaling across different life domains.

*Rhodobacter capsulatus* was historically classified as a non-sulfur bacterium, meaning low or no ability to oxidize sulfide to sulfur. However, it has been noticed that *R. capsulatus* possesses an ability to grow with sulfide as an electron donor (39). In proteobacteria, the initial step of sulfide oxidation is catalyzed by an SQR and flavocytochrome *c*-sulfide dehydrogenase (FCSD), with these two enzymes contributing to polysulfide production (19, 40). *Rhodobacter capsulatus* encodes both an active type I SQR (17), which is essential for sulfide-dependent photoautotrophic growth (41) and is regulated by SqrR (31), and a putative FCSD (rcc03294). In some bacteria, polysulfides are synthesized not only via sulfide oxidation but also via the transsulfuration pathway. Recently, it was shown that an operon regulated by the a polysulfide-responsive transcription factor (CstR) encoding a multidomain sulfurtransferase (CstA), a persulfide dioxygenase PDO (CstB) and a type II SQR, impacts cellular levels of organic thiol persulfides in *Staphylococcus aureus* (30, 42). Moreover, in *Xylella fastidiosa*, the dual domain PDO-rhodanese fusion β-lactamase-like hydrolase (Blh) (42), is projected to convert an organic thiol persulfide to the corresponding thiol and sulfite in response to elevated polysulfide that is sensed by the SqrR ortholog BigR (43). Interestingly, *R. capsulatus* encodes two dual domain ETHE1-sulfurtransferase fusion proteins that appear similar to CstB (rcc02976) and Blh (rcc01824), respectively (38). The other likely polysulfide producing pathway, CAT/MST, found in *E. coli* and mammals (44), is also present in the *R. capsulatus* genome. Thus, *R. capsulatus* seems to possess all the key elements of the polysulfide synthesis network that would enable the understanding of the interplay between polysulfides speciation and the regulation of sulfur-induced intracellular signaling, reinforcing its value as a model organism. To elucidate the contribution of each enzyme to intracellular polysulfide metabolism and signaling, we analyzed polysulfide metabolism-related genes that are regulated by SqrR in *R. capsulatus*. We show that two polysulfide-metabolizing enzymes, SQR and a sulfurtransferase (rhodanese), impact polysulfide-induced transcription and speciation of intracellular sulfane sulfur that in turn modulates the polysulfide response in this organism.

## Results

### Identification of polysulfide-metabolizing enzymes related to polysulfide signaling

To identify polysulfide-metabolizing enzymes that contribute to the SqrR-related polysulfide sensing in cells, we utilized previous RNA-seq transcriptomic data of *R. capsulatus* WT and Δ*sqrR* acquired in the absence and presence of exogenous sulfide (31). We searched these data for candidate enzymes that are projected to play a role in polysulfide metabolism and found five candidate proteins: a candidate peroxiredoxin (rcc00528), SQR (rcc00785), a sulfurtransferase (rcc01181), a rhodanese domain protein (rcc01557), and an uncharacterized flavin- and pyridine nucleotide-dependent disulfide reductase (rcc02679). Transcript levels of these genes were more than 10-fold up-regulated by both treatments with exogenous sulfide and the disruption of *sqrR* (Tables S1 and S2). Although peroxiredoxins are well known to detoxify hydroperoxides such as H_2_O_2_ by its thiol peroxidase activity (45), the sulfenylated peroxidative cysteine can be persulfidated by H_2_S and thus may function in transsulfuration (46). Sulfurtransferase and rhodanese domain proteins are known to traffic persulfide sulfur atoms and thus may be involved in polysulfide metabolism (47, 48). Finally, flavin- and pyridine nucleotide-dependent disulfide reductase, such as glutathione reductase and the closely related thioredoxin reductases, working jointly with various thioredoxins have been shown to be involved in the reduction of proteome persulfide species (6, 49, 50).

**Fig. 1.**
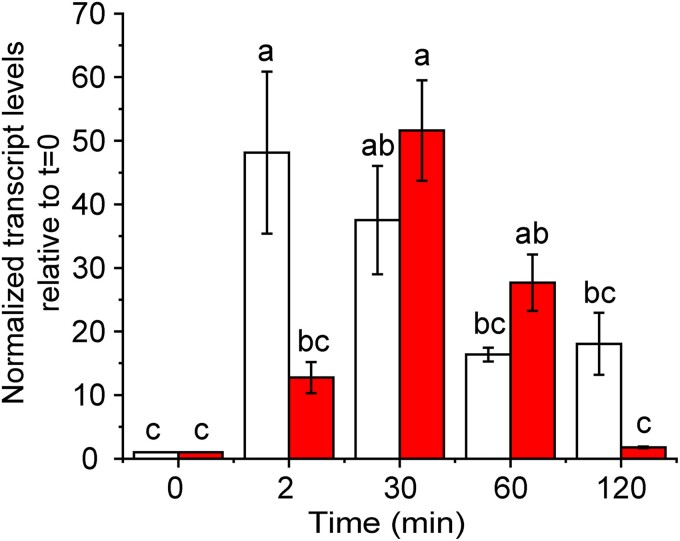
Responsiveness of SqrR-regulated genes to sulfide and polysulfides. Temporal changes in the relative transcript level of the rcc01557 gene assayed by qRT-PCR after treatment with sulfide (*t* = 0 min) in WT (*white* bars) and Δ*sqr* (*red* bars) strains. Cells were grown to the mid-log phase under aerobic conditions, and 0.2 mM sodium sulfide was added at *t* = 0. Data shown are mean ± SE from three biological replicates (*error bars*). Means followed by different letters are significantly different (Tukey test, *P* > 0.05).

To examine the effect of each of these proteins on transcription-based polysulfide signaling, we produced deletion mutants and monitored the expression levels of the SqrR-regulated genes. SqrR represses transcription by binding to the promoter region of the target gene, and is dissociated from the promoter region by forming an intramolecular tetrasulfide bond between two cysteine residues when incubated with GSSH (31). Since SqrR regulates *sqr* and the rhodanese domain protein rcc01557 in response to sulfide stress (Figure S1), we measured the transcript levels of *sqr* gene in the mutants as a proxy for the impact of these enzymes on the SqrR-mediated polysulfide-induced transcription. In the *sqr*-deletion mutant (Δ*sqr*), the rcc01557 transcript was measured in its place. After the treatment with exogenous sodium sulfide, the WT strain shows a rapid increase of sqr and rcc01557 transcript levels, followed by a gradual decrease which is non-significant at later time points (Fig. 1 and Figure S2). Strains harboring mutants of three of the candidate genes (rcc00528, rcc01181, and rcc02679) showed a transcription pattern similar to that of WT strain (Figure S2). In striking contrast, the Δ*sqr* strain showed a significant decrease after 30 min in target gene expression at longer time points (Fig. 1) while rcc01557-deletion mutant (Δ01557) showed sustained and high-level expression of *sqr* after treatment with exogenous sulfide (Figure S2). These data reveal that the loss of SQR and rcc01557 impacts SqrR-mediated polysulfide-induced transcription in precisely opposing ways. We confirmed that recombinant rhodanese domain protein encoded by rcc01557 gene possesses thiosulfate sulfurtransferase activity using thiosulfate and cyanide (CN^−^) as a sulfane sulfur donor and acceptor, respectively (Figure S3). However, the physiological substrate for rcc01557 remains unknown since we could not identify the cognate sulfane sulfur acceptor of this protein. On the other hand, SQR is a major polysulfide producer in bacteria and mitochondria (18, 19) and is essential for sulfide-dependent growth in *R. capsulatus* (41). Therefore, we focused on the function of SQR in the SqrR-mediated polysulfide-induced transcription.

### Polysulfide dynamics in the polysulfide-induced transcription via SQR

**Fig. 2.**
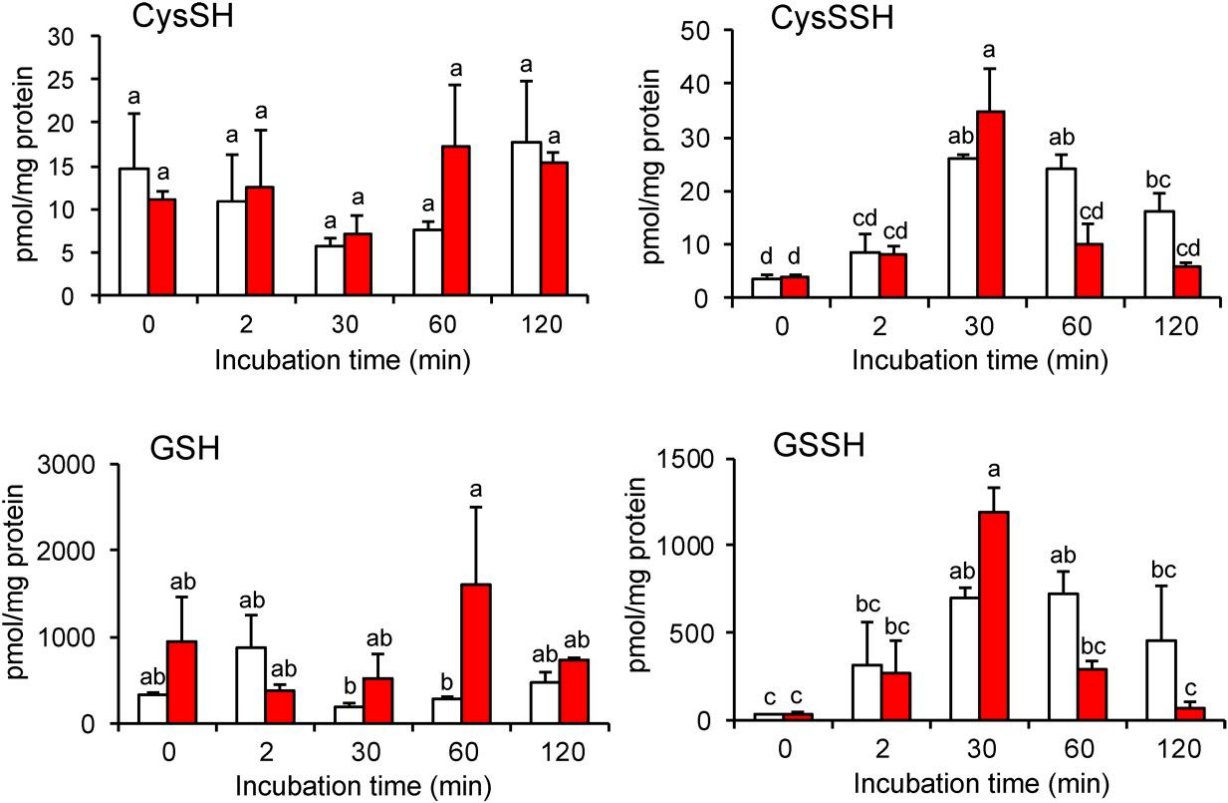
Polysulfide metabolomics *in vivo* in WT (white bars) and Δ*sqr* (red bars). Cells were grown to the mid-log phase under aerobic conditions, and 0.2 mM sodium sulfide was added at *t* = 0. Cells were harvested at each time point and assayed for quantification of various sulfide species. Endogenous production of CysSSH and GSSH were identified by means of HPE-IAM labeling and LC-MS/MS analysis in the bacterial cells. Data are means ± SD (*n* = 3). Means followed by different letters are significantly different (Tukey test, *P* > 0.05).

We quantified the intracellular polysulfide levels in WT and Δ*sqr* strains via LC-MS/MS analysis with β−(4-hydroxyphenyl)ethyl iodoacetamide (HPE-IAM) as the electrophilic trapping agent to measure the changes of cellular polysulfides upon exogenous sulfide/polysulfide exposure, and the contribution of SQR activity to these levels. We first quantified intracellular polysulfides of *R. capsulatus* strains treated with exogenous sulfide under aerobic growth conditions as function of growth time. Endogenous levels of CysSSH, GSSH, and other inorganic polysulfides (e.g. thiosulfate, and inorganic dihydropolysulfide species) were all significantly elevated after treatment with exogenous sulfide, with GSSH the most abundant organic hydropersulfide species detected (Fig. 2 and Figure S4). Reduced glutathione (GSH) is known as the single most prevalent thiol in most organisms (0.1–10 mM) (51) and bacterial intracellular GSH and CysSH were estimated 10–300 μM and 6 μM, respectively, in *E. coli* (52) or 2 mM and 15 μM, respectively, in *Salmonella* Typhimurium (53). In *R. capsulatus*, we estimated the intracellular GSH and CysSH concentration using average cell volume (0.593 μm^3^) (54) and protein concentration per cell (115.5 mg/mL) to be 0.05–2 mM and 2.5–10 μM, respectively. Therefore, our data are broadly consistent with the literature for some but not all Gram-negative organisms. GSSH is clearly the most abundant organic persulfide. In the WT strain, all organic and inorganic polysulfide peaked at around 30 min after treatment with sulfide and remained elevated at the 120 min time point, albeit following a gradual decrease from the 30 min time point (Fig. 2 and Figure S4). In Δ*sqr*, CysSSH, GSSH, and thiosulfate levels were equal to or significantly higher than in WT strain at 30 min but exhibited a rapid decrease after 60 min to a level consistently below that of the WT strain. This initial rapid increase in RSSH has also been observed in Δ*sqr* strains of the pathogen *S. aureus* (19). Moreover, at the 60 min time point, thiols are elevated in Δ*sqr* with a decrease in polysulfides relative to the WT strain at the same time point. Given that SQR is induced by SqrR in response to persulfides and catalyzes RSSH production from RSH, these results indicate that SQR contributes to the endogenous production of RSSH in a way that appears to sustain high levels of organic and inorganic polysulfide at longer time points.

To better understand its contribution to the generation of the intracellular polysulfide pool, we biochemically characterized *R. capsulatus* SQR. For *in vitro* studies, we successfully purified the His-tagged recombinant *R. capsulatus* SQR noncovalently bound with flavin adenine dinucleotide (FAD) (Figure S5). To characterize the kinetics of GSSH and CysSSH formation by SQR, we first measured the steady-state kinetics of SQR utilizing CN^−^ as the S^0^ acceptor to determine available quinone that may function as an electron acceptor for this enzyme. We examined water-soluble ubiquinone-1 (UQ-1) and menadione (MD). Although the steady-state activity was higher with MD, the activity with UQ-1 was also sufficient for further analyses (Figure S6A and B, Table 1). Although UQ-10 is a major quinone in *R. capsulatus* (55) its insolubility prevents biochemical analysis; therefore, we utilized the water-soluble UQ-1 for subsequent measurements. *R. capsulatus* SQR showed activity with both CysSH or GSH (Figure S6C and D) similarly to human SQR (18). While the *V*_max_ values were similar for CysSH or GSH as the S^0^ acceptor, the *K*_m_ values differed ≈2-fold, for a *k*_cat_/*K*_m_ ≈2-fold higher for GSH as the acceptor (Table 2). These data suggest that SQR contributes to the cellular production of both CysSSH and GSSH in *R. capsulatus* and further suggest that the lower levels of CysSSH and GSSH at 60 min post addition of exogenous sulfide in the Δ*sqr* strain may well be due to a lack of endogenous polysulfide production by SQR. Interesting, the inorganic polysulfides, hydrogen disulfide, HS_2_^–^ and hydrogen trisulfide, HS_3_^–^, show the same general trends (Fig. 2 and Figure S4), a finding consistent with the ability of SQR to use inorganic sulfur species as both substrate and S^0^ acceptor (56) or a rapid scrambling and interconversion that tends to characterize these species under physiological conditions (57).

**Table 1.** Sulfide:quinone reductase activity of SQR with sulfide as the substrate in the presence of different quinones as the electron acceptor. 100 nM SQR was reacted with 100 μM ubiquinone-1 or menadione, 4 mM KCN and Na_2_S ranging from 0 to 640 μM.

Electron acceptor	*K*_m_ for sulfide (μM)	*V*_max_ (μmol min ^−1^ μmol ^−1^)	*k*_cat_ */K*_m_ (M^−1^ s^−1^)
Ubiquinone-1	186 ± 43	234 ± 22	(2.1 ± 0.5) × 10^4^
Menadione	45 ± 11	95 ± 6	(3.5 ± 0.9) × 10^4^

**Table 2.** Sulfide:quinone reductase activity of SQR with sulfide as the substrate in the presence of different S^0^ acceptors. 100 nM SQR was reacted with 100 μM ubiquinone-1, 4 mM Cys or GSH and Na_2_S ranging from 0 to 640 μM.

S^0^ acceptor	*K*_m_ for sulfide (μM)	*V*_max_ (μmol min ^−1^ μmol ^−1^)	*k*_cat_ */K*_m_ (M^−1^ s^−1^)
Cys	72 ± 9	48 ± 2	(1.1 ± 0.1) × 10^4^
GSH	38 ± 5	61 ± 2	(2.7 ± 0.4) × 10^4^

Because the temporal patterns of the intracellular polysulfide levels seem to correlate with the temporal changes in transcript level of SqrR-regulated genes (compare Figs. 1 and 2), we suggest that there may be a positive feedback loop between SqrR-mediated polysulfide transcription induction and SQR. To test this hypothesis and identify which polysulfides makes a predominant contribution to this regulation, we investigated if adding polysulfides directly to growing cultures rescued the lower polysulfide-induced transcription observed at longer time points in the Δ*sqr* strain (Fig. 1). To accomplish this, we added an equimolar mixture of sodium sulfide with either cystine or GSSG to the growth medium and measured changes in transcription. Although we confirmed that CysSSH or GSSH were indeed synthesized in a PYS medium subjected to a similar treatment, the concentrations of each were low and other polysulfides were clearly present, with most of the sulfane sulfur present as organic trisulfides (Figure S7). We treated Δ*sqr* with sulfide, cystine-derived polysulfides, and GSSG-derived polysulfides under aerobic growth conditions and the transcriptional changes were measured as a function of growth time. An elevation of rcc01557 transcript was observed at 60 min when cells were treated with exogenous cystine-derived polysulfides (Figure S8A) but not with GSSG-derived polysulfides; this suggests that exogenous cystine-derived polysulfides may be better able to sustain an SqrR-dependent sulfide-response modulated by SQR. Although part of this distinct cellular response to cystine-derived polysulfides and GSSG-derived polysulfides may be attributable to different cellular uptake efficiencies or stabilities of these hydropersulfides in the culture medium, the presence of SQR and/or exogenous cystine-derived polysulfides specifically is clearly capable of enhancing the steady-state lifetime of all polysulfide species in cells (Fig. 2, Figures S4 and S9). We further measured the time-course of the activity of the *sqr* promoter with an *R. capsulatus* WT strain containing *sqr* promoter*-lacZ* fusion following addition of sulfide, cystine-derived polysulfides, and GSSG-derived polysulfides (Figure S8B). The responsivity to GSSG-derived polysulfides was detectably weaker than that of sulfide and cystine-derived polysulfides under aerobic growth conditions (Figure S8B). To explore the effect of redox state under different growth conditions on polysulfide response and to avoid oxidation of CysSSH and GSSH, cells were also grown anaerobically and were treated with polysulfides under these conditions. The activity of the *sqr* promoter activity was also detectably lower when cells were treated with GSSG-derived relative to cystine-derived polysulfides like that found under aerobic conditions (Figure S8C). Interestingly, exogenous sulfide treatment did not induce the activation of the *sqr* promoter under anaerobic conditions revealing that oxidation of exogenous sulfide could play an important role in this process. It is known that chemical oxidation of sulfide with oxygen leads to polysulfide production (58) and that superoxide dismutase (SOD) catalyzes the oxidation of sulfide to produce hydrogen polysulfide (59). These reaction mechanisms may be functional in this bacterium, which may impact physiology in various ways.

To further investigate how exogenous treatment with polysulfides impacts sulfane sulfur speciation inside cells and ultimately elicits a transcriptional response, we measured the intracellular thiols and polysulfide levels in WT cells under these conditions. Treatment with cystine-derived polysulfides shows a transient increase in both cysteine and CysSSH, with CysSSH peaking at two minutes post-induction, falling abruptly, but remaining elevated relative to uninduced cells (Figure S9). At longer time points, all other persulfides remain elevated relative to uninduced cells (Figure S9). In striking contrast, addition of GSSG-derived polysulfides results in no change in glutathione levels, and only transient changes in the other polysulfides that tend to peak at 30 min and generally fall to pre-induction levels at 120 min (Figure S9). These temporal trends in cellular polysulfide levels observed upon cystine-derived vs. GSSG-derived polysulfides treatment bear striking resemblance to what is observed when the WT vs. Δ*sqr* strains are compared (Fig. 2, Figure S4). These two findings taken together suggest that the bolus of CysSSH observed at *t* = 2 min upon treatment (Figure S9) is rapidly sensed by SqrR, which gives rise to high cellular SQR, which in turn maintains polysulfides at an elevated level.

### The reactivity of SqrR toward CysSSH and GSSH

**Fig. 3.**
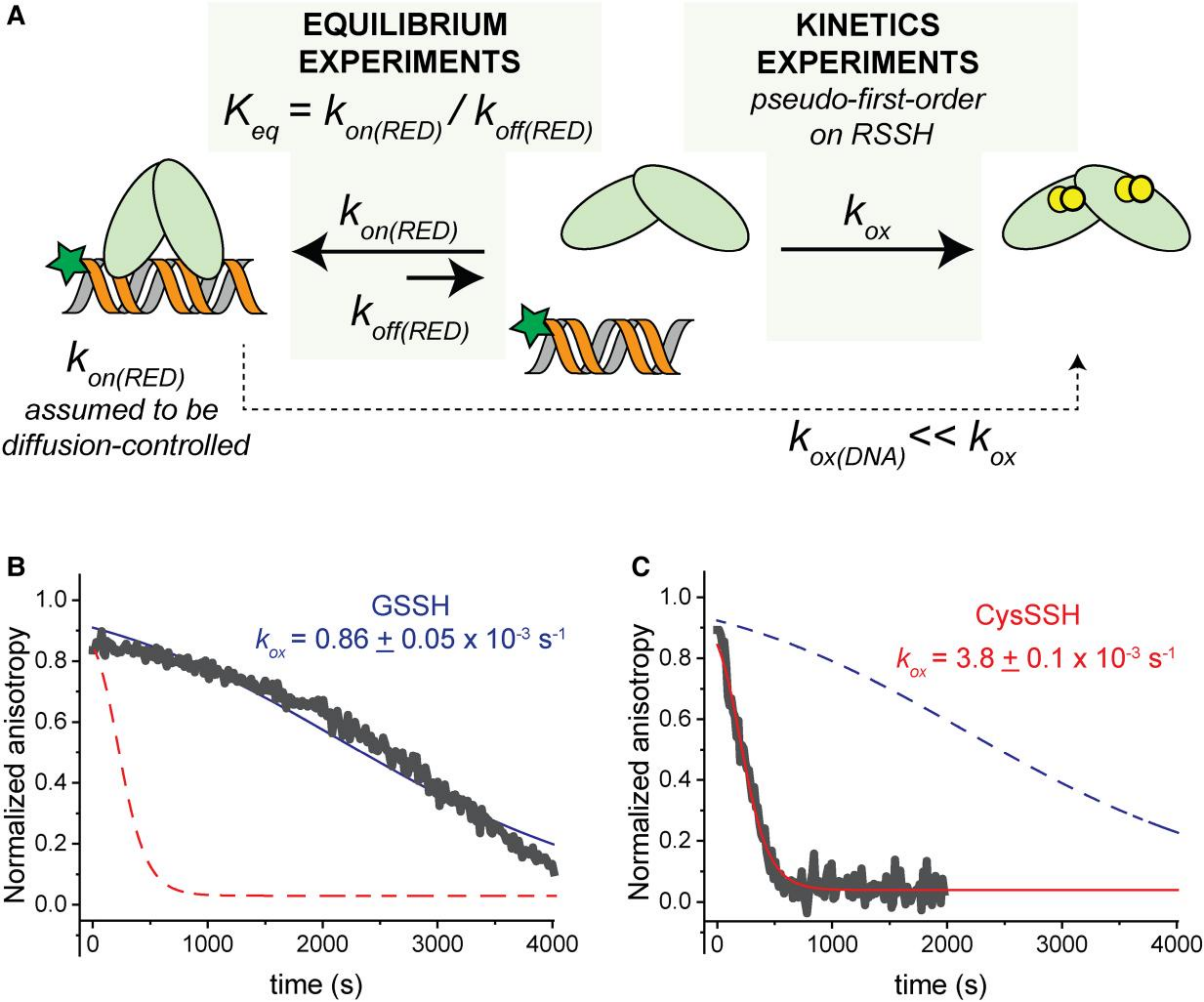
(A) Schematic representation of the fluorescence anisotropy approach used to study the kinetics of the DNA dissociation with RSSH and the experimental constraints. Kinetics of SqrR-DNA dissociation followed by anisotropy upon addition of *in situ*-prepared GSSH (B) or CysSSH (C). Data shown here (*gray* line) corresponds to the representative anisotropy changes after addition of the oxidant. The *blue* and the *red* lines correspond the fit of the data to simple pseudo-first order rate of oxidation (*k*_ox_) with the parameters shown according to the model depicted in panel A. The dashed lines represent the fitting with the parameters corresponding to the oxidant not shown.

The temporal coupling of sulfane sulfur speciation and SqrR-mediated transcriptional regulation makes the prediction that CysSSH can induce an SqrR transcriptional response very rapidly (*t* ∼ 2 min). Although the rates at which distinct organic persulfides react with the same thiol have not been reported (60, 61), CysSSH may be intrinsically more reactive towards SqrR thiols than other RSSH. To test this, we examined the intrinsic reactivity of free SqrR or SqrR-DNA complexes toward CysSSH vs. GSSH prepared and analyzed as described (Table S3). We previously showed that SqrR forms an intramolecular tetrasulfide crosslink between two Cys residues (C41, C107) upon treatment with GSSH *in vitro* (31, 35); CysSSH was not examined in those studies. We turned to fluorescence anisotropy as a robust reporter of *in vitro* DNA binding (35). We performed these experiments using a C9S SqrR variant and a fluorescein-labeled oligonucleotide harboring to the rcc1451 operator to ensure tight binding, while minimizing the complications from oxidative chemistry of the non-conserved Cys9 (35). We titrated SqrR to saturation (Figure S10), and after addition of 20-fold excess of sulfane sulfur from *in situ* generated persulfides (GSSH or CysSSH) (Table S3) (36) over SqrR thiol, monitored the decrease in anisotropy which reports on the dissociation of SqrR from the DNA (Fig. 3). Direct SqrR titrations reveal that the rate constant of the reduced protein binding to DNA is too fast to be measured, as the final value of anisotropy after each addition is reached in less than 30 s and a single time point anisotropy measurement requires 20 s (Figure S10). Nevertheless, the direct titration of the protein allows us to determine the DNA-binding constant of the reduced form in the absence of TCEP. Strikingly, the rates of DNA dissociation upon persulfide addition differ significantly between GSSH and CysSSH (Fig. 3). We fit this dissociation kinetics to a simple model of protein oxidation that captures the difference between each treatment, which shows that the rate of oxidation with GSSH is about four times slower than for CysSSH (Fig. 3A). In both cases, a new equilibrium is ultimately reached, which allowed for an estimation of DNA-binding affinities of the GSSH- and CysSSH-oxidized forms. These were comparable and ≈100-fold lower than that of the reduced protein (Table 3). After this new equilibrium condition was reached, SqrR can be reduced upon addition of TCEP which restores DNA-binding, revealing that oxidation is readily reversible in both cases (Figure S10C and D). Overall, these two experiments (Fig. 3, Figures S8–S10) are internally consistent and suggest that SqrR-mediated de-repression of transcription may occur more rapidly in the presence of CysSSH relative to GSSH.

**Table 3.** Equilibrium and kinetics parameters for DNA binding and dissociation upon oxidation for SqrR C9S.

	*K_eq_* [x 10^5^ M^−1^]^a^	*k_ox_* [x 10^−3^ s^−1^]^a^	
		GSSH	CSSH
SqrR reduced	270 ± 25 (*n* = 6)	0.86 ± 0.05 (*n* = 3)	3.8 ± 0.1(*n* = 3)
SqrR, GSSH treated	2.5 ± 0.5 (*n* = 3)	—	—
SqrR, CysSSH treated	1.8 ± 0.8 (*n* = 3)	—	—

a Errors correspond to standard deviations of the mean for n number of experiments carried out under the same conditions: 25 mM HEPES, pH = 7.0, 400 mM NaCl, 1 mM EDTA, 25°C. The treatment with GSSH or CysSSH corresponds to a 20-fold addition of sulfane sulfur relative to the protein subunit concentration.

**Fig. 4.**
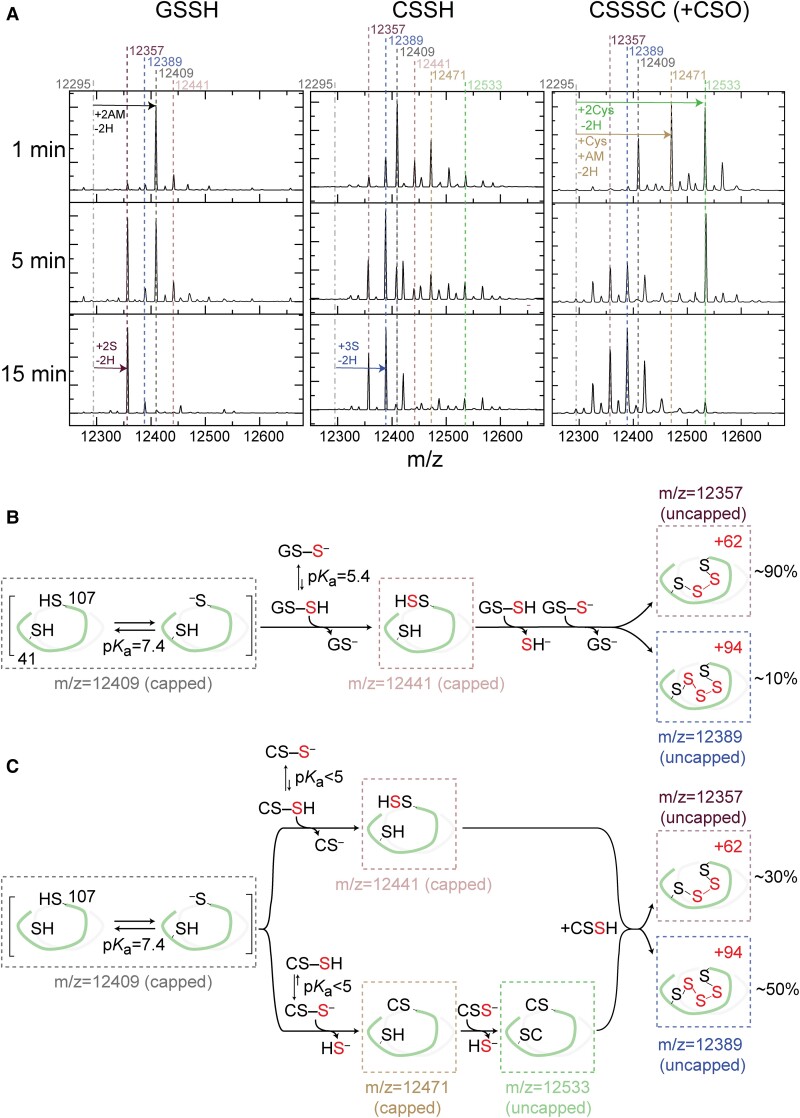
(A) Representative kinetic traces that illustrate the time-course of reactivity of C9S SqrR toward various oxidants under various conditions. LC-ESI-MS spectra of C9S SqrR using a 20-fold excess of glutathione persulfide (GSSH), cysteine persulfide (CysSSH), each prepared as described (Table S3), and impure cystine trisulfide (CysSSSCys + CysSO), for variable times followed by addition of excess IAM to cap both thiols and persulfides. Solution conditions: 30 μM protomer C9S SqrR, 150 mM phosphate buffer pH 7.4, 1 mM EDTA. Vertical dashed lines depict the intact masses (amu) of the different oxidation states of the SqrR protomer (12295, reduced, dot-dashed *black*; 12357, tetrasulfide, *purple*; 12389, pentasulfide, *blue*; 12409, AM capped reduced, *black*; 12471, AM capped persulfide, *salmon*; 12441, AM capped and mixed disulfide with Cys, *tan*; 12533, two mixed disulfides with Cys, *green*). Summary of intermediates and final products with mechanistic proposal compatible with the results for GSSH (B) and CSSH (C) treatment. The p*K*_a_ of the different thiols are from prior reports (35, 68).

To test this idea, we exploited an MS-based assay to identify the modification of C9S SqrR by a both GSSH and CysSSH in a time-resolved manner under anaerobic conditions. Interestingly, the product distributions with CysSSH and GSSH are distinct (Fig. 4) despite the fact that the sulfane sulfur species composition in each mixture is virtually identical, dominated (≈89%) by authentic CysSSH or GSSH in each case (Table S3). GSSH induces the formation of a predominantly tetrasulfide crosslink between C41 and C107 as observed previously (31, 35), while CysSSH gives rise to a mixture of products, with a C41–C107 pentasulfide bridge dominating the product species (Fig. 4). This result is reminiscent of the chemistry we observed previously with cysteine trisulfide (CysSSSCys) doped with small amounts of Cys sulfoxide (CSO). CysSSSCys reacts readily with SqrR only in the presence of CSO, a strong electrophile. (Fig. 4) (35). These data clearly show that SqrR forms mixed disulfides with Cys and those intermediates tend to be on pathway to the formation of longer (*n* ≥ 2 S atoms) S bridges (Fig. 4C). Overall, these reactivity assays show that CysSSH reacts more rapidly and with a distinct mechanism and product distribution relative to GSSH (Fig. 4B-C).

## Discussion

This study demonstrates a significant coupling between endogenous polysulfide production and polysulfide-regulated gene transcription. We have identified two polysulfide-metabolizing enzymes, SQR and a rhodanese, which contribute to SqrR-mediated polysulfide-induced transcription via polysulfide production and transsulfuration. SQR can catalyze H_2_S-dependent persulfidation of Cys and GSH producing CysSSH and GSSH, respectively (Figure S6 and Table 2) and is primarily responsible for sustaining cellular polysulfide levels, since Δ*sqr* shows only an early phase accumulation of these species (Figs. 1 and 2). These features of the Δ*sqr* strain can be rescued by supplementation with extracellular cystine-derived polysulfides (Figure S8A). We also show that SqrR-DNA complex possesses a higher reactivity toward CysSSH relative to GSSH (Fig. 4). Altogether, our results suggest that the SqrR-mediated transcription is subjected to feedback regulation by SQR.

Polysulfides can be produced non-catalytically, from the reaction of sulfide with a disulfide bonded or sulfenylated organic thiols (62) and catalytically, via oxidation by SQR and FCSD (19, 40). In *R. capsulatus*, SQR clearly catalyzes polysulfide formation (Table 2) and transcription of *sqr* is repressed by SqrR in the absence of sulfide, whereas the transcription of gene encoding FCSD is not controlled by SqrR (31). Although polysulfides are also synthesized via the transsulfuration pathway, our previous RNA-seq data showed that the other candidate sulfurtransferases, such as the PDO (rcc02976), a PDO-rhodanese fusion protein (rcc01824) and CAT/MST, are not regulated in response to sulfide by SqrR (31); therefore, these enzymes may be involved in constitutive polysulfide homeostasis in *R. capsulatus*. Indeed, our transcriptomics data showed an increase in polysulfides in Δ*sqr* at 30 min, and a decrease at 60 and 120 min (Fig. 1). This suggests that while polysulfides can be produced independently of SQR, the speciation of these polysulfides may vary in response to SQR-independent production facilitating their oxidation and, ultimately, efflux. Although the mechanism is not known it seems likely that the rhodanese encoded by rcc01557 is involved in sulfane sulfur trafficking. Here, only SQR and rhodanese, whose expression is controlled by sulfide, impacted polysulfide-induced transcription (Fig. 1 and Figure S1). The identified rhodanese has thiosulfate sulfurtransferase activity (Figure S3) and SQR catalyzes the generation of CysSSH and GSSH from the corresponding thiols as S^0^ acceptors (Figure S6 and Table 2). Disruption of these genes caused altered responsivity in the late phase of polysulfide-induced transcription (Fig. 1 and Figure S2). Consequently, our observations suggest that these sulfurtransferases contribute to the polysulfide-induced transcriptional response via polysulfide production and/or interconversion of polysulfides, which may well be rapid under physiological conditions.

Interestingly, the ratios for CysSSH to CysSH and GSSH to GSH reveal that these organic persulfides accumulate to a level that is comparable to that the corresponding thiol (approaching ≈50% at intermediate time points). Typically, in mammals, 5–20% of cysteine pool and 0.5–1.5% of glutathione pool were observed under unstressed conditions (21). Indeed, in *R. capsulatus*, CysSSH and GSSH levels are ≈20 and 0.1% of each total thiol before treatment with sodium sulfide (Fig. 2). It is known that beta- and gammaproteobacteria, especially *Allochromatium vinosum,* intracellularly accumulates hydrophobic sulfur globules (63). Moreover, the sulfur globules accumulate to 25% of the overall volume of the cell in *A. vinosum* (64). Given that the sulfur globules are produced via sulfide oxidation by SQR and identified as cyclooctasulfur (S_8_), a covalently closed polysulfide (63), it is not surprising that SQR-mediated sulfide oxidation produces large amounts of polysulfides in bacteria. In *A. vinosum*, three different hydrophobic sulfur globule proteins, SgpA, B and C, are required for the formation of sulfur granules (65, 66), and *R. capsulatus* does not have these does not have these proteins. It is unknown how intracellular polysulfide formation studied here impacts the accumulation of extracellular sulfur globules known to form when *R. capsulatus* is grown on sulfide; this might be traced to the possibility that some SQR localizes to the periplasm (41).

GSSH is the most prevalent among various hydropersulfide derivatives in the cells (6). In both the bacterium *Salmonella* and in the mouse, the amount of GSSH was 5–30-fold higher than that of HSSH and CysSSH under normal growth conditions (21, 53). In *R. capsulatus* the concentrations of GSSH vs. CysSSH differ by 10–30-fold both with and without exogenous sodium sulfide treatment (Fig. 2). Therefore, despite the 4-fold higher oxidation rate of SqrR by CysSSH compared to GSSH (Fig. 3), as the enzymatic production of CysSSH and GSSH by SQR would appear to be quite similar (Table 2), it is not clear under which conditions SqrR can be selectively induced by CysSSH. Moreover, Fig. 4 indicates that both CysSSH and GSSH can form an intramolecular polysulfide linkage between residues C41 and C107 of the recombinant SqrR protein. The differences in chemical properties that would elicit a faster and sustained transcriptional response of SqrR are unknown, but the acidity of Cys is lower than GSH, making Cys a better leaving group (67), which may result in more rapid oxidation. Moreover, the structure of the reduced form and diamide adduct of SqrR suggests that the cavity containing the two reactive Cys can be easily blocked by an initial nucleophilic attack by one of the thiols (35). This steric effect has been shown to play a role in selectivity of persulfides relative to other electrophiles (35) and may well be responsible for selectivity for CysSSH over GSSH *in vitro*. This is particularly so when one considers that CysSSH can form additional intermediates such as mixed disulfides that are not observed in the reaction with GSSH (Fig. 4). While this difference in reactivity may not fully explain what is seen in cells given the difference in concentration between GSSH and CysSSH, it does provide valuable insights into what could be determining selectivity as it is likely that SqrR-DNA complexes preferential reactivity towards LMWT or protein hydropersulfides can explain the fast (<2 min) transcriptional response.

**Fig. 5.**
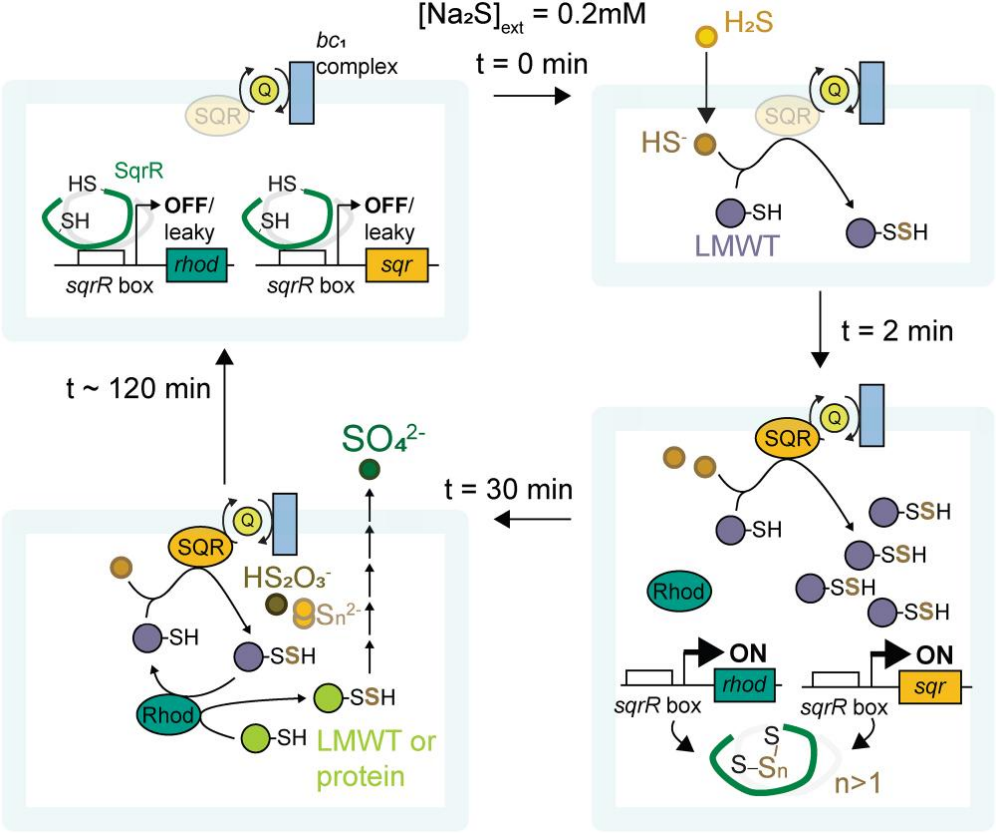
Schematic of how polysulfides impact SqrR-mediated polysulfide-induced transcription for wild-type cells stressed with Na_2_S. Exogenous or endogenous sulfide is quickly (*t* < 2 min) converted to polysulfides via a “housekeeping” or constitutive response as a result of chemical and/or enzymatic processes. The so-generated polysulfides react with SqrR and the repressor activity of SqrR is maintained in an inactive DNA-binding state by tetrasulfide formation (*n* > 1) between the Cys residues (2 min < *t* < 30 min). During this time, the expression of genes encoding SQR and the rhodanese (Rhod) are de-repressed and high levels of low molecular weight thiol (LMWT) and proteome persulfides are continuously generated from sulfide via an “inductive response” (*t* > 30 min). After this time, other more oxidized sulfur species (S_n_^2–^, thiosulfate and sulfate) begin to accumulate, thus reducing cellular [RSSH], restoring unstressed physiology and SqrR-mediated transcriptional repression (*t* ∼ 120 min).

In mitochondria, CysSSH is mainly synthesized from cysteine regardless of exogenous sulfide by CARS2, which is a more likely source of biological persulfides and leads to subsequent formation of GSSH via persulfide scrambling (6, 21). In *R. capsulatus*, exogenous cysteine-derived polysulfides induce a rapid and transient increase in CysSSH which then falls precipitously with the concomitant and subsequent formation of other polysulfides (Figure S9); similar effects by CysSSH generated from exogenous cystine also occurs in the mitochondrion (6). Therefore, we suggest that CysSSH and other hydropersulfides more reactive than GSSH function as the signaling molecules that induce of SqrR-regulated transcription at early times, and sustained prolonged steady-state lifetimes of a number of polysulfides, thus sustaining SqrR-mediated polysulfide-induced transcription (Fig. 5 and Figure S11).

In summary, SqrR-mediated polysulfide transcriptional regulation is modulated by both constitutive and inductive responses (Fig. 5 and Figure S11). When cells are exposed to exogenous or endogenous sulfide, polysulfides are generated rapidly by chemical and/or enzymatic reactions, and SqrR losses the ability to repress transcription. The subsequent expression of SQR leads to sustained levels of polysulfides, which prevents SqrR reduction, DNA re-association and transcriptional repression. Although not investigated in detail here, sustained levels of polysulfides may have an as yet unknown physiological function; however, cells generally must protect themselves from excess polysulfides. In other organisms, thioredoxin and glutaredoxin can independently reduce inorganic polysulfides and protein persulfides (24). Moreover, sulfurtransferases and persulfide dioxygenases may also serve as “off-switches” to runaway polysulfide production. Indeed, our results suggest the possibility that the rhodanese may reduce persulfide-oxidized SqrR via intermolecular transsulfuration either directly or indirectly, restoring transcriptional repression and thus avoiding SQR-derived polysulfide levels from continuing to rise. These findings demonstrate how sulfur signaling flows from polysulfide metabolism to transcriptional regulation and back again. Further elucidation of the functional role(s) of other polysulfide metabolic enzymes promises a better understanding of how these processes impact physiological functions in this bacterium and other organisms.

## Materials and methods

The Materials and methods are described in Supplementary material, Materials and Methods. They include bacterial strains, growth conditions, qRT-PCR, β-galactosidase assay, purification of recombinant SqrR and SQR, sulfurtransferase assay, fluorescence anisotropy and mass spectrometry-based sulfuromics analysis. All primers used in this research are listed in Table S4. Data represent the mean of at least three independent experiments (error bars indicate SE of the mean). The *P*-value and statistical significance of difference were analyzed by using unpaired *t*-tests (*P* < 0.05, significant).

## Supplementary Material

pgad048_Supplementary_Data

## Data Availability

All data are presented within the manuscript or are available in the Supplementary Materials.
