# Supplementary material for: Polysulfide metabolizing enzymes influence SqrR-mediated sulfide-induced transcription by impacting intracellular polysulfide dynamics
Source: PNAS Nexus. 2023 Feb 10;2(3):pgad048. doi: 10.1093/pnasnexus/pgad048 (PMC9995734; doi:10.1093/pnasnexus/pgad048)
Supplement: pgad048_Supplementary_Data [file pgad048_supplementary_data.docx]

**Supplementary Information for**

Polysulfide metabolizing enzymes influence SqrR-mediated sulfide-induced transcription by impacting intracellular polysulfide dynamics

Takayuki Shimizu, Tomoaki Ida, Giuliano T. Antelo, Yuta Ihara, Joseph N. Fakhoury, S hinji Masuda, David P. Giedroc, Takaaki Akaike, Daiana A. Capdevila and Tatsuru Masuda

Takayuki Shimizu

Email: ctshimizu@[g.ecc.u-tokyo.ac.jp](mailto:xxxxx@xxxx.xxx)

**This PDF file includes:**

Supplementary text

Figures S1 to S11

Table S1 to S4

SI References

SI Materials and Methods.

**Bacterial strains, media, and growth conditions**

*R. capsulatus* WT strain SB1003 and mutant strains were grown under aerobic or anaerobic-light (photosynthetically) conditions at 30°C in PYS medium (1) as described (2, 3). Illumination was provided by a light-emitting diode (λ_max_ = 850 nm) (CCS) for photosynthetic growth. For anaerobic growth, cultures in screw-capped test tubes were almost completely filled with the medium. Gentamycin, rifampicin and spectinomycin were used at a concentration of 1.5 µg/mL, 75 µg/mL and 10 µg/mL, respectively.

*Escherichia coli* strains were grown in Luria Bertani (LB) medium at 37°C. Kanamycin, gentamycin and spectinomycin were used at a concentration of 50 µg/mL, 10 µg/mL and 40 µg/mL, respectively.

**Cloning and mutagenesis**

Two ~500-bp DNA fragments consisting of N-terminal and C-terminal regions of each gene were amplified by polymerase chain reaction (PCR) with KOD One polymerase (TOYOBO). Two sets of primer pairs were used for the amplification; one is a forward primer (sqr_F1, rcc00528_F1, rcc01181_F1, rcc01557_F1 and rcc02679_F1) and a reverse primer (sqr_R1, rcc00528_R1, rcc01181_R1, rcc01557_R1 and rcc02679_R1), and the other is a forward primer (sqr_F2, rcc00528_F2, rcc01181_F2, rcc01557_F2 and rcc02679_F2) and a reverse primer (sqr_R2, rcc00528_R2, rcc01181_R2, rcc01557_R2 and rcc02679_R2). The sequence of each primer are shown in Table S4. *Sph*I, *Eco*RV and *Bam*HI restriction sites were designed at additional polynucleotide tails, respectively. The first PCR fragment was digested with *Sph*I and *Eco*RV and the second PCR fragment was digested with *Bam*HI and *Eco*RV. After the digestion, these two fragments were mixed and ligated together with the *Sph*I-*Bam*HI-cut pZJD29a (4). The obtained plasmids were introduced into *R. capsulatus* WT cells by conjugation with the mobilizing *E. coli* strain S17-1/*λpir*, as described (4). Cells undergoing a single crossover event were selected by plating exconjugants on PYS plates containing gentamycin and rifampicin. After sequential cultivation in the absence of antibiotics, sucrose-resistant but gentamycin-sensitive cells were selected on PYS-agar plates containing 5% sucrose to generate mutants. A deletion was confirmed by PCR amplification followed by sequencing analysis.

**RNA Isolation and Quantitative Real-Time PCR (****qRT-PCR)**

*R. capsulatus* was grown aerobically to mid-log phase in PYS medium. For sulfide treatment, a final 0.2 mM of Na_2_S or CysSSH or GSSH was added and cells were further grown for 0, 30, 60, 90 and 120 min. At each time point, 0.5 ml of cells were harvested with total RNA of each sample extracted using NucleoSpin RNA (TaKaRa). A typical OD_260_ to OD_280_ ratio of RNA sample was approximately 2.0. Reverse transcription was performed using PrimeScript RT Reagent kit (TaKaRa). cDNA was amplified using THUNDERBIRD Next SYBR qPCR Mix (TOYOBO). Signal detection and quantification were performed in duplicate using the MJ Mini Thermal Cycler and MiniOpticon Real-Time PCR System (Bio-Rad). As an internal control, the house-keeping gene *rpoZ* that encodes DNA-directed RNA polymerase omega subunit was used with the gene-specific primers (Table S4). In this experiment, treatment with GSSH or CysSSH were established by addition of the mixture with an equal molar excess of freshly dissolved Na_2_S and glutathione disulfide or cystine.

**Preparation of isotope-labeled internal standards ([d4]-HPE-IAM adducts)**

Various stable isotope-[d4]-labeled HPE-IAM adducts with CysSH/CysSSH, GSH/GSSH, and HS^-^/HS_2_^-^/HS_3_^-^/HS_2_O_3_^-^ were synthesized according to our previous report (6, 7). In brief, 0.5 mM cysteine and GSH was mixed with 0.5 mM NaHS in the presence of 0.5 mM propylamine NONOate [(2-hydroxy-1-methyl-2-nitrosohydrazino)-N-methyl-1-propanamine] (Dojindo Laboratories) in 10 mM Tris-HCl buffer (pH 7.4) at room temperature for 5 min, after which 5 mM [d4]-HPE-IAM (Toronto Research Chemicals Inc.) was added to the reaction mixture, and then the mixtures were incubated at room temperature for 20 min. HS^-^/HS_2_^-^/HS_3_^-^ and HS_2_O_3_^-^ adducts with [d4]-HPE-IAM were synthesized as follows. NaHS, Na_2_S_2_, Na_2_S_3_ and Na_2_S_2_O_3_ (0.1 mM respectively) were reacted with 0.5 mM [d4]-HPE-IAM in 10 mM Tris-HCl buffer (pH 7.4) at room temperature for 20 min. These [d4]-labeled HPE-IAM adducts were purified by using the HPLC system under the following conditions: YMC-Triart C18 column (50 ×2.0 mm inner diameter, 3 μm); flow rate: 0.2 mL/min; mobile phase A: 0.1% formic acid; mobile phase B: 100% methanol; gradient: linear gradient (from 5% to 90% B, for 15 min); wavelength detector (set at 275 nm); temperature: 40°C. The recovered isotope-labeled standards were concentrated using the Speed Vac Concentrator and then dissolved in 0.1% formic acid. The HPLC-purified products were identified as stable isotope-labeled HPE-IAM adducts by their HPLC-retention time and mass spectrometry analysis.

**Determination of concentrations of various HPE-IAM adduct standards**

To determine the concentrations of GSH- and GSSH-HPE-IAM adducts (standards), for example, different concentrations of HPE-IAM solutions (in 25 mM Tris-HCl pH 8.0) were prepared. These solutions were reacted with an excess amount of GSH and followed by injection into an HPLC and monitored for absorption at 275 nm. After confirming that all peak of HPE-IAM was converted to that of the GSH-HPE-IAM adduct, a calibration curve for the concentration of GSH-HPE-IAM adduct was prepared from the peak area of this HPE-IAM adduct. On the other hand, the standards of HPE-IAM adducts with GSSH and higher polysulfides that were purified by HPLC were injected into the HPLC and their concentrations were calculated from the calibration curve of GSH-HPE-IAM adduct, drawn as just mentioned. The concentrations of other sulfur HPE-IAM adducts were similarly calculated from calibration curves, in which HPE-IAM was reacted with excess amounts of various sulfur-containing metabolites, like cysteine, NaHS, and Na_2_S_2_O_3_. To rigorously confirm the validity of the methodology with GSSH-HPE-IAM adduct, it was reduced to get free GSH using TCEP immobilized on agarose CL-4B (Merck), after which the eluate was reacted again with HPE-IAM to form GSH-HPE-IAM adduct. The concentration of GSH-HPE-IAM thereby generated was quantified by HPLC in the same manner as above, and in turn was correlated to that of authentic GSSH-HPE-IAM. Meanwhile, the concentrations of various stable isotope-labeled HPE-IAM adducts, were determined by LC-MS/MS, by using known concentrations of respective HPE-IAM adducts, as being spiked to the HPLC as internal standards for LC-MS/MS *vice versa* as internal standards.

**Polysulfide quantification**

*R. capsulatus* WT and *sqr*-deletion mutant grown to the mid-log phase under aerobic conditions were treated with 0.2 mM sodium sulfide for 0-120 min, after which they were harvested at each time point and washed with cold PBS for 3 times. The intracellular polysulfide level was quantified according to our previous reports (5, 6). In briefly, the methanol extract of the cells containing 5 mM HPE-IAM were incubated at 37 °C for 20 min. After centrifugation, aliquots of the supernatants of the lysates were diluted 12 times with 0.1% formic acid containing known amounts of isotope-labeled internal standards, which were then analyzed via LC-ESI-MS/MS for polysulfide determination. A triple quadrupole (Q) mass spectrometer LCMS-8060 (Shimadzu) coupled to the Nexera UHPLC system (Shimadzu) was used to perform LC-ESI-MS/MS. RSS-HPE-IAM adducts were separated by means of Nexera UHPLC with a YMC-Triart C18 column (50 × 2.0 mm inner diameter) under the following elution conditions: mobile phases A (0.1% formic acid) with a linear gradient of mobile phases B (0.1% formic acid in methanol) from 5 to 90% for 15 min at a flow rate of 0.2 ml/min at 40 °C. Various polysulfide derivatives were identified and quantified by means of multiple reaction monitoring.

**Overexpression and purification of SqrR, SQR and rhodanese**

SqrR WT and the C9S variant were purified as previously described (7, 8). The SQR and rhodanese overexpression plasmids were constructed as follows. At first, DNA fragment encoding full-length *sqr* was amplified by PCR with a forward primer, pCold::sqr_F and pCold::1557_F, and a reverse primer, pCold::sqr_R and pCold::1557_R. The amplified DNA was cloned into *Nde*I-cut pColdI vector (TaKaRa) by In-Fusion HD Cloning kit (Clontech). The obtained plasmid was transferred into *E. coli* strain BL21 (DE3) and the recombinant protein was overexpressed by induction with 0.2 mM isopropyl-β-D-thiogalactopyranoside (IPTG) at 16°C overnight (12-16 h).

For purification of SqrR and rhodanese, cells in a 500 mL culture were harvested and resuspended in 20 mL nickel column loading buffer composed of 20 mM Tris-HCl (pH 8.0), 500 mM NaCl, 5 mM imidazole and 10% glycerol, and lysed by sonication. The lysate was clarified by centrifugation at 30,000 x*g* for 30 min at 4°C. The resultant supernatant was passed through a 45 µm membrane filter (Millipore) and loaded onto a 1-mL HisTrap column with an ÄKTAprime (GE Healthcare), and washed with 30-column-volume of the wash buffer containing 20 mM Tris-HCl (pH 8.0), 500 mM NaCl, 20 mM imidazole, and 10% glycerol. His-tagged proteins were eluted with a gradient of 20 mM imidazole to 500 mM imidazole in the loading buffer over a 15-column-volume total. In the case of SqrR, the SUMO-tag was proteolytically cleaved by UlP1 protease and tag-less SqrR was then isolated bypassing through a 1-mL HisTrap column. Purified tag-less SqrR and His-tagged rhodanese were dialyzed with a buffer containing 20 mM Tris-HCl (pH 8.0), 500 mM NaCl and 6% Glycerol. Protein concentration was measured by the Bradford assay.

For purification of SQR, cell pellet was resuspended with 25 mM Tris-HCl (pH 8.0), 500 mM NaCl, 20 mM imidazole, 10% glycerol and 2 mM Tris(2-carboxyethyl)phosphine (TCEP), and lysed by sonication. The centrifuged and filtrated lysate was loaded onto a 1-mL HisTrap column with an ÄKTAprime (GE Healthcare), and washed with 30-column-volume of the wash buffer containing 25 mM Tris-HCl (pH 8.0), 500 mM NaCl, 50 mM imidazole, 10% glycerol and 2 mM TCEP. His-tagged SQR was eluted with a gradient of 50 mM imidazole to 500 mM imidazole in the loading buffer over a 15-column-volume total. Purified SQR was stored at −80°C until they were used with a final glycerol concentration of 20% (v/v) added. When they were used for measurement of enzymatic activity, TCEP was removed by ultrafiltration using the degassed buffer composed of 25 mM Tris-HCl (pH 8.0), 500 mM NaCl and 10% glycerol under anaerobic conditions to obtain the reduced SQR without reducing agent. Protein concentration was measured by the Bradford assay.

Absorption spectra of purified SQR was recorded from 300 nm to 600 nm using a Ultrospec 2100 pro UV/visible spectrophotometer (Amersham Biosciences) for analysis of FAD binding. To verify noncovalent binding between FAD and SQR protein, the purified SQR was treatment of with 5% trichloroacetic acid and fluorescence emission spectra of the supernatant and the resuspended pellet were recorded using a FP-6200 fluorescence spectrophotometer (Jasco) The samples were excited at 365 nm and the emission spectra were recorded from 470 to 650 nm.

**Sulfurtransferase and SQR enzymatic activity**

The sulfurtransferase activity was measured spectrophotometrically to detect the iron-thiocyanate formation using thiosulfate and potassium cyanide as sulfur donor and acceptor, respectively (9). Reaction mixtures contained 5 μM rhodanese, 5 mM KCN and thiosulfate ranging from 0 to 150 mM, 20 mM Tris-HCl (pH 8.0), 500 mM NaCl, 6% glycerol, in a volume of 500 μl. These enzymatic reactions were initiated via the addition of thiosulfate and the mixtures were incubated at 30°C for 30 min. 250 μl of 15% formaldehyde was added to quench the reaction, and color was developed by the addition of 750 μl of ferric nitrate reagent [100 g of Fe(NO_3_)_3_.9H_2_O and 200 ml of 65% HNO_3_ per 1,500 mL]. Thiocyanate (complexed with iron) was quantified by measuring the absorbance at 460 nm and interpolating the value on a standard curve for potassium thiocyanate.

SQR activity was measured spectrophotometrically as a detection of sulfide-dependent quinone reduction. Reaction mixtures contained 100 nM SQR, 100 μM ubiquinoe-1 (UQ-1) or menadione (MD), and 4 mM KCN and Na_2_S ranging from 0 to 640 μM, buffered by degassed 25 mM Tris-HCl (pH 8.0) and 200 mM NaCl, in a volume of 500 μl. These enzymatic reactions were initiated via the addition of Na_2_S and the mixtures were incubated at 30°C for 5 min. Ubiquinol-1 and menadiol were quantitated by *A*_280_-*A*_300_ nm using ε = 17,800 M^-1^ cm^-1^ (10) and *A*_270_-*A*_295_ nm using ε = 14,800 M^-1^ cm^-1^ (11), respectively. To measure the catalytic activity of SQR over sulfide in the presence of 4 mM Cys or GSH, the same assay was used with UQ-1 as the electron acceptor.

**β-galactosidase assay**

*R. capsulatus* strain containing the *sqr* promoter region and *lacZ* fusion plasmid as described (7) was grown aerobically or photosynthetically to mid-log phase in PYS medium. For sulfide induction, a final 0.6 mM of Na_2_S or CysSSH or GSSH was added and cells were grown further for 0, 30, 60, 90 and 120 min. After the induction, 10 mL of cells were harvested, and β-galactosidase activity was determined essentially as described previously (12). Final results were obtained as the amount of o-nitrophenyl-β-D-galactopyranoside (ONPG) hydrolyzed per min per mg protein. In this experiment, treatment with GSSH or CysSSH were established by addition of the mixture with an equal molar excess of freshly dissolved Na_2_S and glutathione disulfide or cystine.

**Binding and kinetic experiments**

The DNA-binding equilibrium of SqrR and the dissociation upon polysulfide treatment were characterized by fluorescence anisotropy. We used a fluorescein modified oligonucleotide based on the sequence of the rcc1451 gene operator, as previously described (8) (see Table S4). We used an automatic injector to titrate up to ~600 nM SqrR into 10 nM fluorescein-labeled rcc1451 in buffer 25 mM HEPES, pH =7.0, 400 mM NaCl, 1 mM EDTA (25.0 °C). After saturation, a 20-fold excess (relative to SqrR concentration) of GSSH or CysSSH was added to the solution, and the anisotropy changes were followed until a new equilibrium condition was reached. Finally, TCEP was added to the cuvette to a final concentration of 5mM to assess reversibility of the oxidation of SqrR. The equilibrium binding constant for the reduced protein was obtained by fitting each titration single site isotherm (8). The equilibrium constants for oxidized SqrR were obtained by calculating the DNA occupancy values in the equilibrium condition after addition of GSSH or CysSSH (n = 3 for each treatment). The kinetics of DNA release after addition of polysulfide were modeled using DynaFit (13), fitting the data to a simple model using 3 rate constants: the *k_on_* and *k_off_* corresponding to the rates of association and dissociation between protein dimer and DNA, and a rate of oxidation of the protein dimer, *k_ox_*. The ratio between *k_on_* and *k_off_* was fixed to the estimated association binding constant *K_eq_* for reduced SqrR (270 x 10^5^ M^-1^, Table 3). The script used in this model is provided below. The rates were estimated using a global fit for the 3 replicates of each treatment.

**Mass spectrometry profiling**

These experiments were performed as previously described (8). Briefly, 30 mM glutathione disulfide and 10 mM oxidized cystine stocks in 150 mM phosphate buffer pH 7.4 were mixed with an equal volume of 150 mM Na_2_S in the same buffer and incubated at 30°C for 30 min in anaerobic conditions to generate the persulfides GSSH and CysSSH, which fraction RSS species quantified, as described more fully below (14). The reaction mixture for the oxidation of SqrR consisted of 30 μM SqrR in 150 mM phosphate buffer pH 7.4 and 600 μM of GSSH or CysSSH in anaerobic conditions. At different timepoints, fractions of the reaction mixtures were taken, and the oxidation was quenched by addition of 60 mM iodoacetamide. Samples were then diluted ~10 fold and MS was performed in the Laboratory for Biological Mass Spectrometry at Indiana University using a Waters Synapt G2S mass spectrometer coupled with a Waters ACQUITY UPLC iClass system. Data were collected and analyzed using MassLynx software (Waters).

**Analysis of sulfane sulfur speciation in *in situ*-generated mixture of CysSSH and GSSH using HPE-IAM and LC-MS**

All steps to generate CysSSH and GSSH were performed as described previously (15). In a typical reaction, 5 mM cystine or GSSG is mixed with 25 mM Na_2_S in 300 mM sodium phosphate pH 7.4 (1 mL total volume) and incubated at 30 °C for 30 min. To determine the total concentration of sulfane sulfur by cold cyanolysis, 25 µL was withdrawn from each mixture and mixed with 20 μL of 1 M ammonium hydroxide, 180 μL milli-Q H_2_O, 25 μL 0.5 M KCN, and incubated at room temperature for 45 min. After this, 5 μL 37% formaldehyde and 50 μL Goldstein reagent were added and the A_460_ determined, with the concentration of sulfane sulfur determined using a standard curve of known [KSCN] (0.5 to 4 mM). 100 μM sulfane sulfur from each mixture was then mixed with 3 mM “heavy” *d*_4_-HPE-IAM in 1% DMSO and samples incubated at 37 °C for 1 h in screw-capped tubes prepared in an anaerobic chamber and filtered using 0.22 μm spin filters. 25 µL was then withdrawn and mixed with 25 µL of a solution containing 100 µM “light” HPE-IAM-derivatived cysteine, glutathione and Na_2_S. 5 µL was routinely subjected to LC-MS on a YMC Triart C18 column as previously described (16). Quantitation of organic per- and polysulfide species and inorganic polysulfide species was determined using the relative peak areas of known concentrations of light thiol and Na_2_S, respectively. This analysis assumes that the ionization efficiencies of per- and polysulfidated thiols and individual S_n_ species are identical to the parent HPE-IAM thiol or di-derivatived HPE-IAM sulfide, respectively. The results of this analysis are shown in Table S3.

**Dynafit Script – CysSSH data:**

[task]

data = progress

task = fit

[mechanism]

P -> PO : kox

PDNA -> DNA + P : koff_red

DNA + P -> PDNA : kon_red

[constants]

kox = 0.000864 ? ; unit: seg-1

koff_red = 3.6

kon_red = 0.027 * koff_red ; diffusional 0.1 nM-1s-1=10^8

[concentrations]

PDNA = 10

[data]

directory

extension txt

file

Anis_AddedCysSSH | response DNA = 0.0156 , PDNA = 0.021 | conc P = 273; concentration of dimer, RSSH = 10926

file Anis_AddedCysSSH_duplicate | response DNA = 0.0158 , PDNA = 0.021 | conc P = 258; concentration of dimer, RSSH = 10300

Anis_AddedCysSSH_triplicate | response DNA = 0.0161 , PDNA = 0.0214 | conc P = 251; concentration of dimer, RSSH = 10022

[output]

directory C:\output_AddedCSSH_1_try

[settings]

{Output}

BlackBackground = n

[end]

**Dynafit Script – GSSH data:**

______________________________________________________________________

[task]

data = progress

task = fit

[mechanism]

P -> PO : kox

PDNA -> DNA + P : koff_red

DNA + P -> PDNA : kon_red

[constants]

kox = 0.02017 ? ; unit: seg-1

koff_red = 3.6

kon_red = 0.027 * koff_red ; diffusional 0.1 nM-1s-1=10^8 M-1s-1

[concentrations]

PDNA= 10

[data]

mesh linear from 1 to 5000 step 1

directory

C:\ Dynafit_Input

extension txt

file 20210929_Anis_AddedGSSH | response DNA = 0.0156 , PDNA = 0.0215? | conc P = 318 ; concentration of dimer, RSSH = 12723

file 20210929_Anis_AddedGSSH_duplicate| response DNA = 0.0156 , PDNA = 0.0215? | conc P = 318 ; concentration of dimer, RSSH = 10300

file 20211004_Anis_AddedGSSH_triplicate | response DNA = 0.0156 , PDNA = 0.0225 | conc P = 251 ; concentration of dimer, RSSH = 10022

[output]

directory C:\ output_AddedGSSH_final

[settings]

{Output}

BlackBackground = n

[end]

**Table S1.** Transcript levels of polysulfide metabolism-related genes affected by sulfide in WT. Data was cited from (7).

| Accession number | Annotation | Fold change (with/without sulfide) | p-value |
| --- | --- | --- | --- |
| rcc00528 | candidate peroxiredoxin | 14.5 | 4.30E-164 |
| rcc00785 | SQR | 17.4 | 4.53E-213 |
| rcc01181 | sulfurtransferase | 32.8 | 1.06E-176 |
| rcc01557 | rhodanese domain protein | 40.5 | 1.46E-234 |
| rcc02679 | uncharacterized flavin- and pyridine nucleotide-dependent disulfide reductase | 17.8 | 2.48E-124 |

**Table S2.** Transcript levels of polysulfide metabolism-related genes affected by loss of SqrR. Data was cited from (7).

| Accession number | Annotation | Fold change (Δ*sqrR*/WT) | p-value |
| --- | --- | --- | --- |
| rcc00528 | candidate peroxiredoxin | 137.7 | 0 |
| rcc00785 | SQR | 57.4 | 0 |
| rcc01181 | sulfurtransferase | 88.4 | 6.70E-167 |
| rcc01557 | rhodanese domain protein | 88.9 | 0 |
| rcc02679 | uncharacterized flavin- and pyridine nucleotide-dependent disulfide reductase | 13.0 | 2.33E-160 |

**Table S3.** Molar fraction of sulfane sulfur species in the GSSH and CysSSH mixtures prepared as described above^a^

| Sulfane sulfur species | GSSH mixture | CysSSH mixture |
| --- | --- | --- |
| RSSH | 0.89 | 0.88 |
| RSSSH | 0.003 | 0.01 |
| S_2_ | 0.11 | 0.10 |
| S_3_+S_4_ | ≤0.01 | ≤0.01 |

^a^Since these reactions generally result in ≈25% yield of sulfane sulfur, the major constituents are thiol and Na_2_S which are unreactive toward thiols (15).

**Table S4.** List of all primers used in this research.

| Name | Sequence 5’-3’ |
| --- | --- |
| sqr_F1 | TTGCATGCGATCGACAACTGGGCGCCCTATTACAT |
| sqr_R1 | TTTTGATATCCACGATATGAGCCATCTGTCCCTCC |
| sqr_F2 | TTTTGATATCGGGCATCGACAAGCTGAAGGCCGTC |
| sqr_R2 | TTGGATCCACGCCCGGCTGATCGTCGAGGCGATCA |
| rcc00528_F1 | TTGCATGCCTTCACCGCGAAGATCGTGATCAATGC |
| rcc00528_R1 | TTTTGATATCCGTCTTGTAGCTCATCTGGGCCTCC |
| rcc00528_F2 | TTTTGATATCCCCACCACCGCCTGAGCGGCGCAAA |
| rcc00528_R2 | TTGGATCCTGCGGATGAACCAGGCCGCCTGTTCCG |
| rcc01181_F1 | TTGCATGCGCGCCGACAGCAGGACCTGACGTTTCA |
| rcc01181_R1 | TTTTGATATCGCGGTCAAGGGTCATCGGAAGCCTC |
| rcc01181_F2 | TTTTGATATCTTCGAGGCGAAATAAACCGATCCCG |
| rcc01181_R2 | TTGGATCCGCCCTTCATGGGGATCTGCATCGGCAT |
| rcc01557_F1 | TTGCATGCCGAGGTCGTCTTCTGCTCGATCGAAAT |
| rcc01557_R1 | TTTTGATATCCAGGAAGTTGAACATCGAAAACTCC |
| rcc01557_F2 | TTTTGATATCGTCGTTCGGGTGTAAGAAAGCGGGG |
| rcc01557_R2 | TTGGATCCATTGCCGGCTGCACCTATTGCACGCCG |
| rcc02679_F1 | TTGCATGCCCCGAGTATTATTTCCGTATCCGCGAC |
| rcc02679_R1 | TTTTGATATCGACGATATGGGTCATGGGGGCTTCC |
| rcc02679_F2 | TTTTGATATCCGGGCGTTCCGTTAAGCCAGGGGCC |
| rcc02679_R2 | TTGGATCCGGATCGCGCTTGAACTCGCCTCGCTGA |
| pCold::sqr_F | TCGAAGGTAGGCATATGGCTCATATCGTGGTTCTGGGTG |
| pCold::sqr_R | GTACCGAGCTCCATACTATCCCTTTTTGACGGCCTTCAG |
| pCold::1557_F | TCGAAGGTAGGCATATGTTCAACTTCCTGCGCTC |
| pCold::1557_R | GTACCGAGCTCCATATTACACCCGAACGACCGCGC |
| qRT-sqr_F | CGCAAGGAAGACAAGGTCAC |
| qRT-sqr_R | CGAGGGCACGAAATGATAC |
| qRT-01557_F | GAAATGCGACCCGTCAAG |
| qRT-01557_R | GAACCACGGGTTTGTCCA |
| qRT-rpoZ_F | GAGATCGCCGATGAAACC |
| qRT-rpoZ_R | TCGTCGACCTCGATCTGG |
| Rcc01451_F_Fluorescein | (5FluorT)-GACATATTCACAACTCGGAATGTAA |
| Rcc01451_R | TTACATTCCGAGTGGTGAATATGTCA |


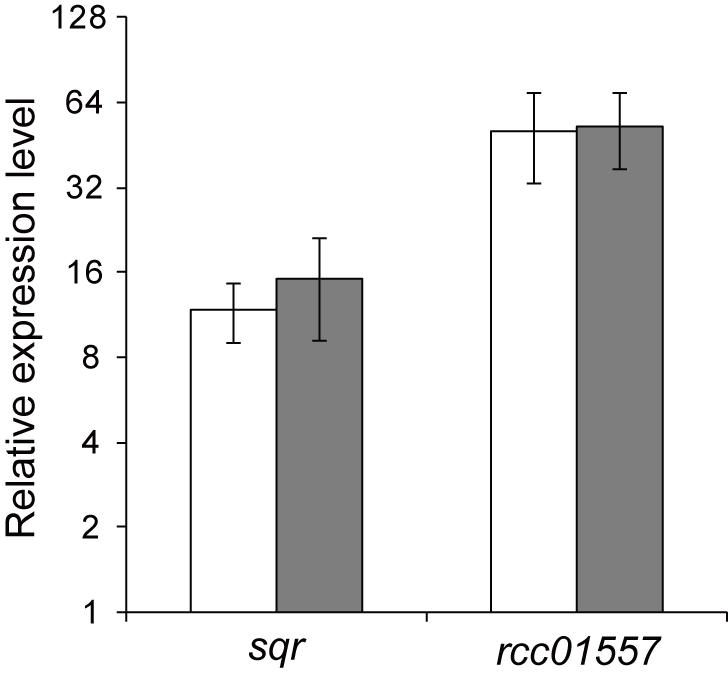


Fig. S1. Transcript levels of *sqr* and rcc01557 relative to untreated WT cells after addition of 0.2 mM sulfide (gray) or without sulfide (white) to *R. capsulatus* Δ*sqrR* cells. Data shown are mean ± S.E. (*error bars*) of 3 experiments. These data establish that SqrR is a repressor of the expression of the *sqr* and rcc01557 genes.


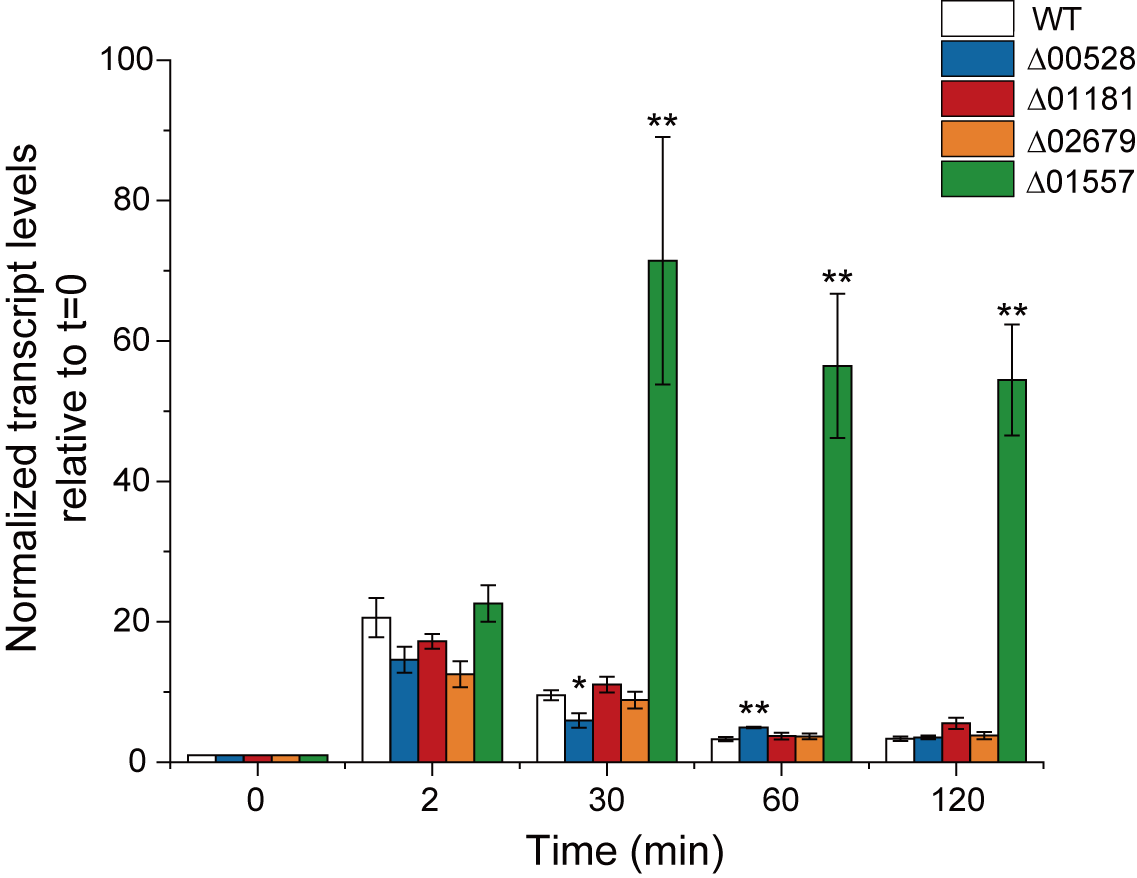


Fig. S2. Change in the relative level of transcripts of *sqr* after treatment with 0.2 mM sulfide added at *t* = 0 min in *R. capsulatus* WT and selected gene-deletion mutants. Data shown are mean ± S.E. (*error bars*) of 3 experiments. Only loss of rcc01557 gives rise to sustained expression of the *sqr* gene. The data were analyzed using a Student’s t-test. The significant level of difference between WT and mutants are indicated by * and ** for *p* <0.05 and *p* <0.01, respectively.


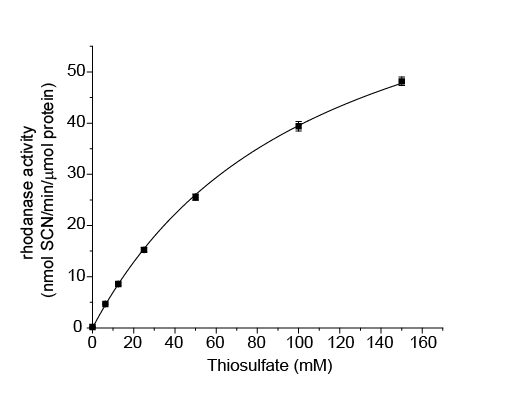


Fig. S3. Kinetic characterization of the thiosulfate sulfurtransferase (TST) activity of the rhodanese encoded by rcc01557. This rhodanese exhibits TST activity and displays Michaelis-Menten kinetics with *K*_m_ of 114 ± 3 mM and *V*_max_ of 85 ± 1 nmol min^−1^ μmol^−1^. Data shown are mean ± S.D. (*error bars*) of 3 experiments.


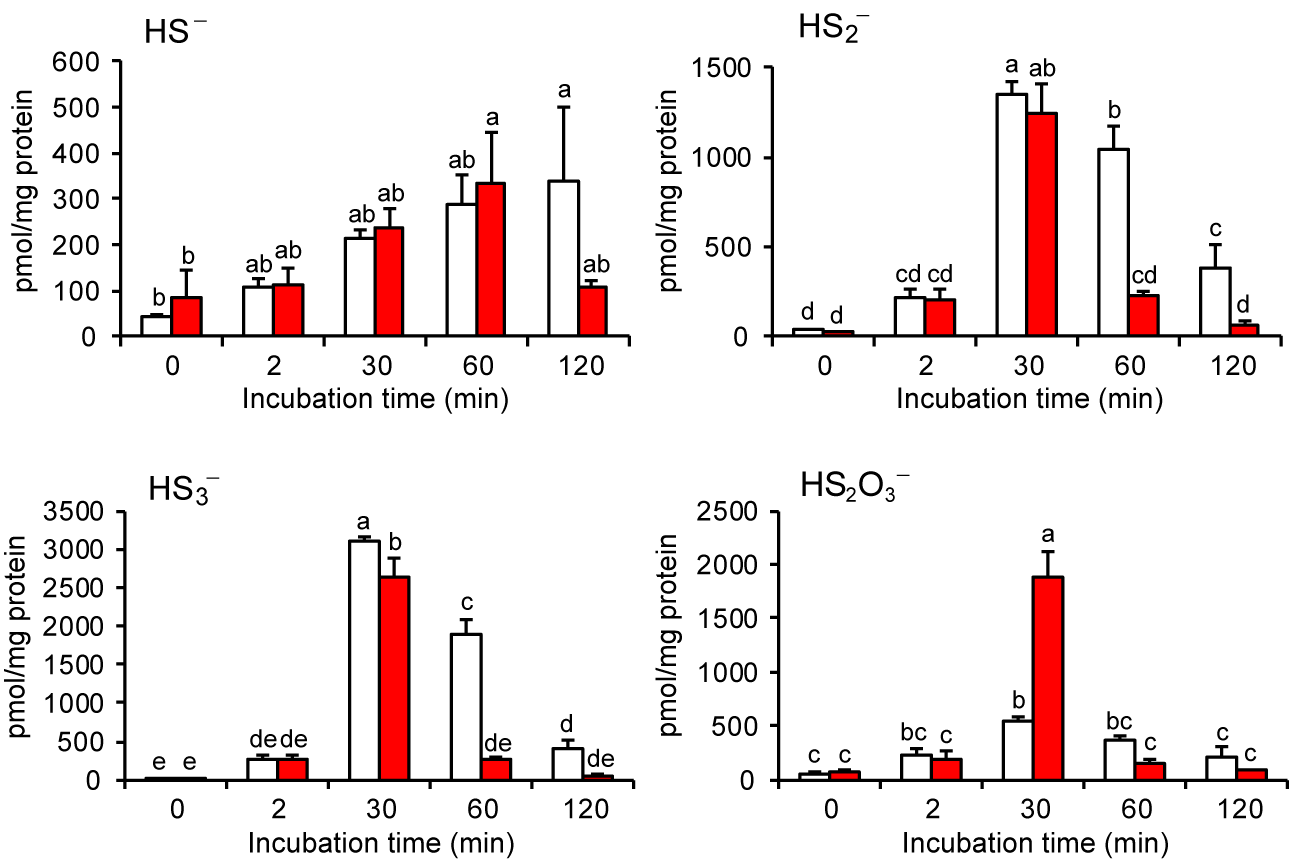


Fig. S4. Inorganic polysulfide metabolomics *in vivo* in WT (*white* bars) and Δ*sqr* (*red* bars) strains. Cells were grown to the mid-log phase under aerobic conditions, and 0.2 mM sodium sulfide was added at *t* = 0. Cells were harvested at each time point and assayed for quantification of various inorganic polysulfide. Endogenous levels of sulfide (HS–) (*upper left*) polysulfides HSS^–^ (*upper right*) and HSSS– (*lower left)* and thiosulfate (*lower right*) were identified by means of HPE-IAM labeling LC-MS/MS analysis in the bacterial cells. Data shown are mean ± S.D. (*error bars*) of 3 experiments. Means followed by different letters are significantly different (Tukey test, p>0.05).


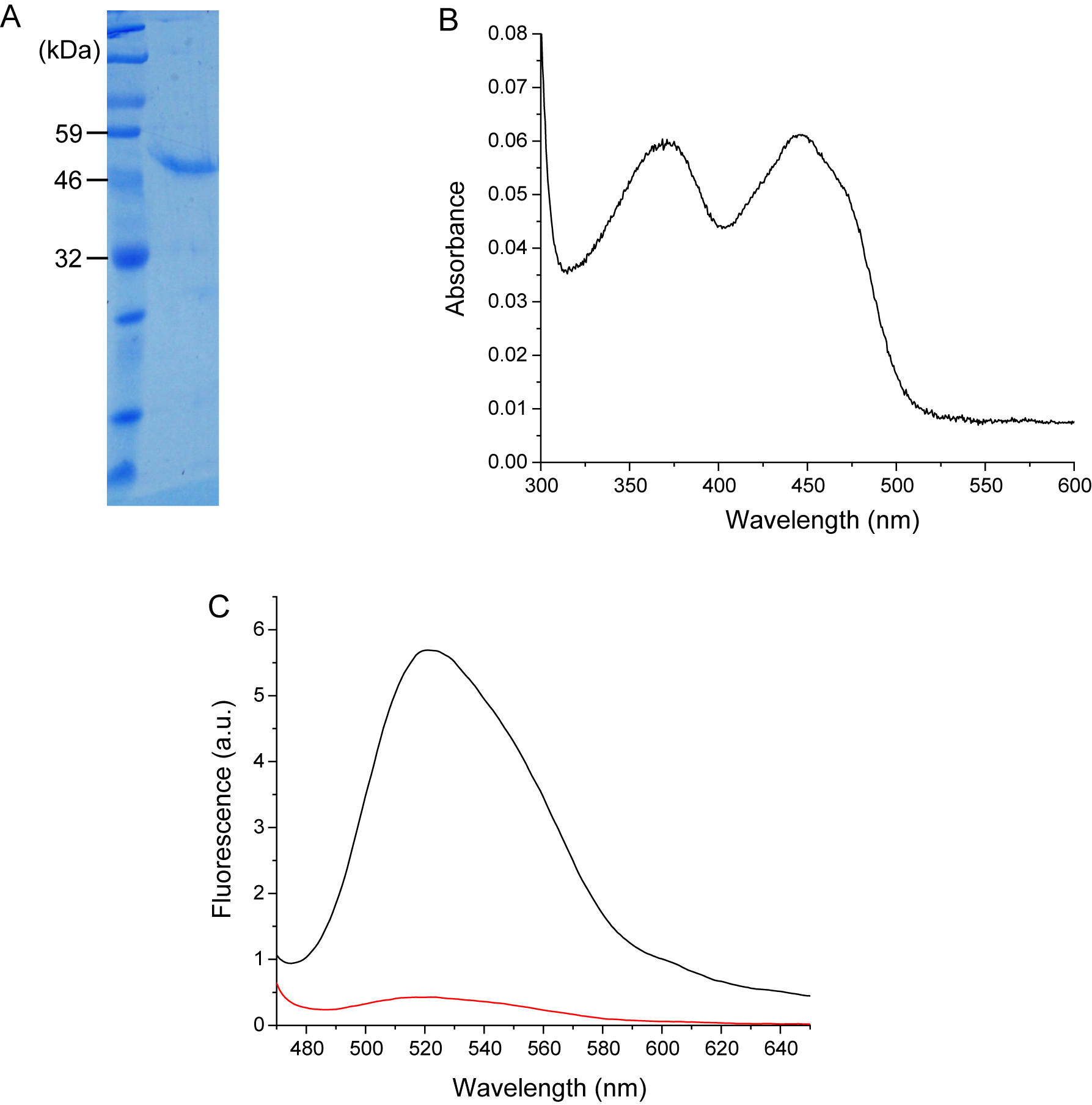


Fig. S5. Biochemical properties of *R. capsulatus* SQR. (A) SDS-PAGE analysis of purified SQR recombinant protein. Purified SQR showed a single band with an apparent molecular weight of 49.3 kDa, calculated from its amino-acid sequence. Molecular weight markers are shown in the left lane. (B) A UV-visible spectrum of SQR under aerobic conditions. (C) A fluorescence emission spectra (excitation at 365 nm) of supernatant (*black* line) and resuspended protein (*red* line) after a brief incubation with 5% trichloroacetic acid at room temperature. The data show that the FAD is not covalently bound to SQR.


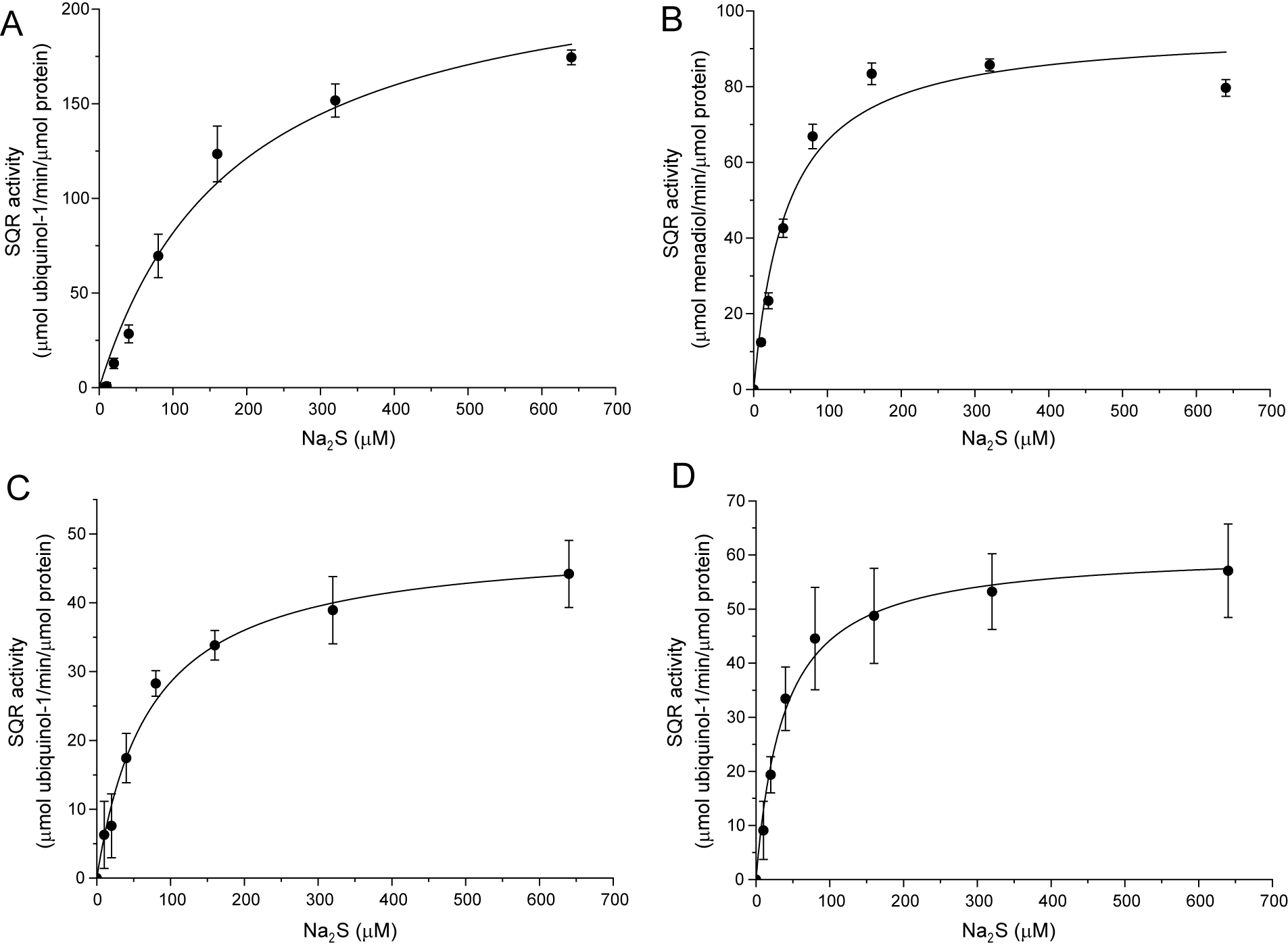


Fig. S6. Kinetic characterization of sulfide oxidation by SQR. (A, B) The SQR activity was measured with cyanide (CN^−^) as a S^0^ acceptor in the presence of ubiquinone-1 (A) or menadione (B) as the electron acceptor. (C, D) The SQR activity was measured with Cys (C) or GSH (D) as a S^0^ acceptor in the presence of ubiquinone-1 as the electron acceptor. Data shown are mean ± S.D. (*error bars*) from 3 experiments


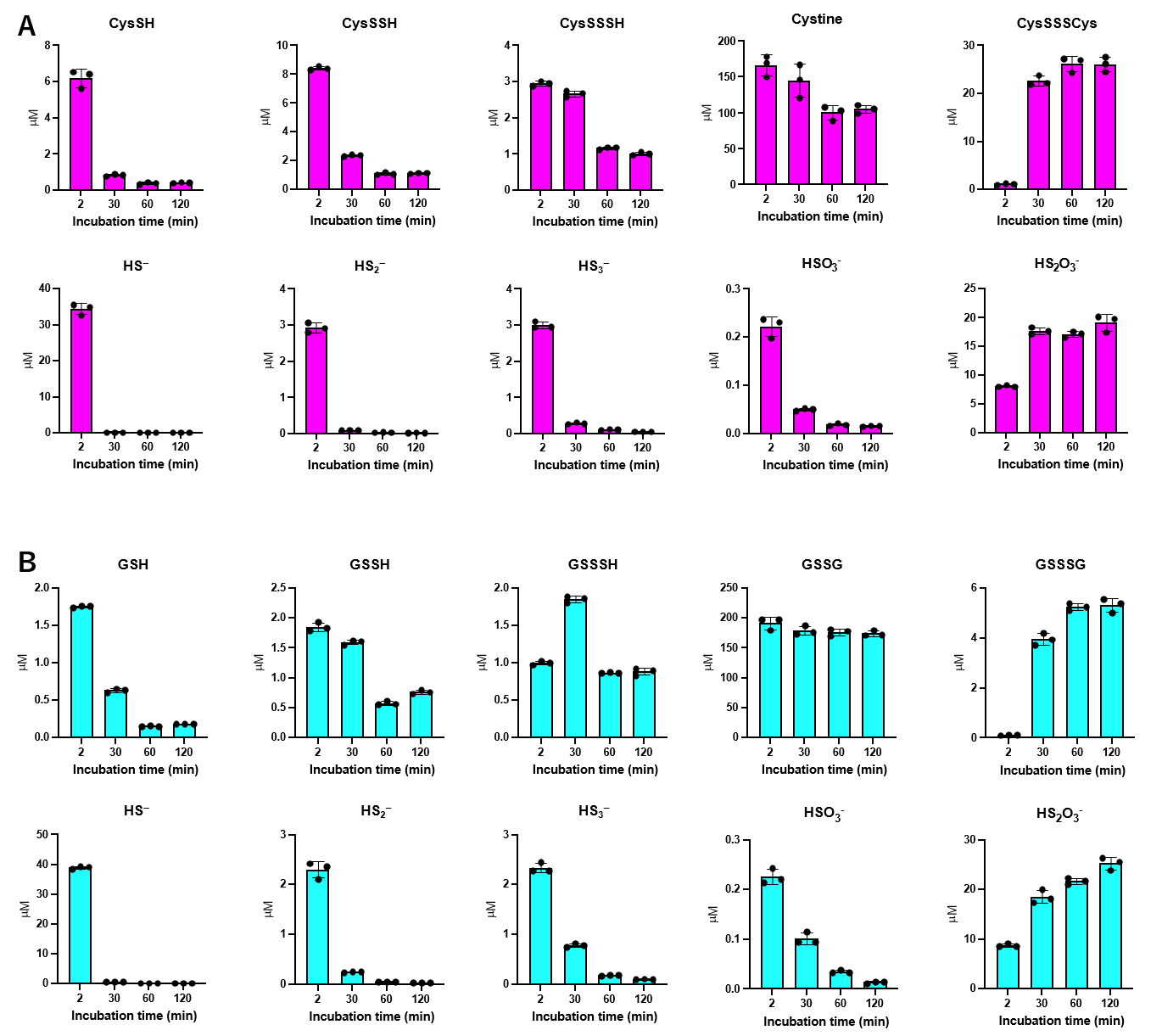


**Fig. S7**. The composition of polysulfide in PYS medium. Data shown are mean ± S.D. (*error bars*) of 3 experiments. Final 0.2 mM cystine (A) or 0.2 mM GSSG (B) was mixed into PYS medium and 0.2 mM Na_2_S.9H_2_O was subsequently added at *t* = 0. Solution was harvested at each time point and assayed for quantification of various inorganic polysulfide.


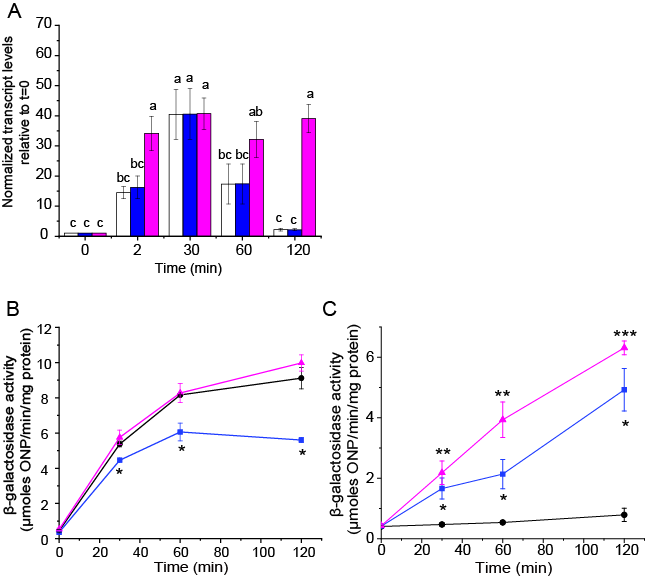


Fig. S8. Responsiveness of SqrR regulated genes to sulfide and polysulfide. (A) Temporal changes in the relative transcript level of the rcc01557 gene assayed by qRT-PCR after treatment with sulfide (t = 0 min) in Δ*sqr* strain. Cells were grown to the mid-log phase under aerobic conditions, and 0.2 mM sodium sulfide (open), GSSH (blue) or CysSSH (magenta) were added at t = 0. Means followed by different letters are significantly different (Tukey test, p>0.05). (B) β-galactosidase activity measurements of the *sqr* promoter region and *lacZ* fusion in WT. Cells were grown to mid-log phase under aerobic conditions and 0.6 mM sodium sulfide (black), CysSSH (magenta) or GSSH (blue) was added at t = 0. Cells were harvested at each time point and assayed for β-galactosidase activity. (C) Same as in (B), except that cells were grown under anaerobic conditions and treated with sulfide and polysulfide anaerobically. Data shown are mean ± S.E. from three biological replicates (error bars). β-galactosidase activity data were analyzed using a Student’s t-test. The significant level of difference between sulfide and polysulfide treatment is indicated by *, **, and *** for p <0.01, p <0.001 and p <0.0001, respectively.


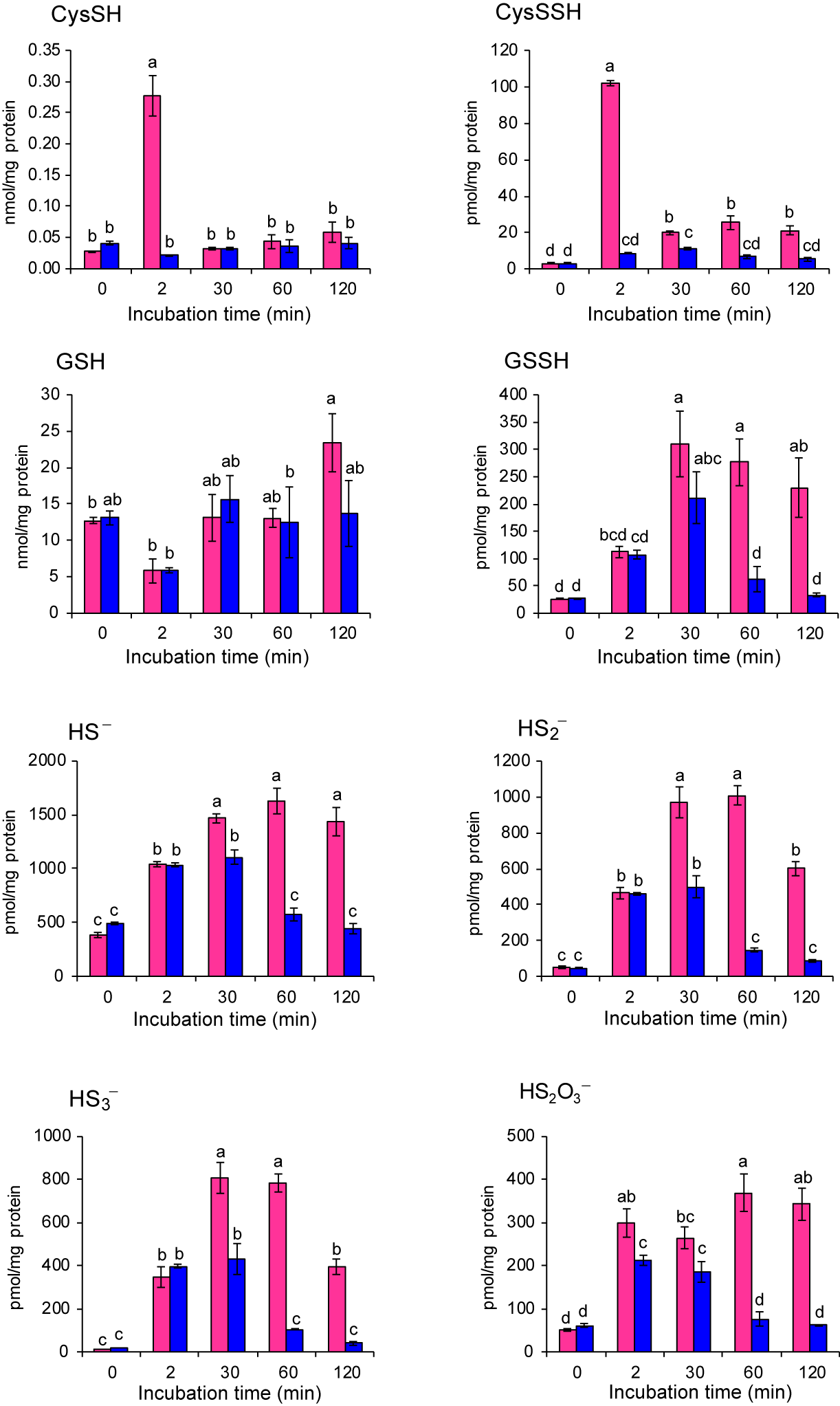


Fig. S9. Inorganic polysulfide metabolomics *in vivo* in WT. Cells were grown to the mid-log phase under aerobic conditions, and 0.2 mM CysSSH (magenta bars) or GSSH (blue bars) were added at *t* = 0, respectively. Cells were harvested at each time point and assayed for quantification of various inorganic polysulfide. Data shown are mean ± S.D. (*error bars*) of 3 experiments. Means followed by different letters are significantly different (Tukey test, p>0.05).


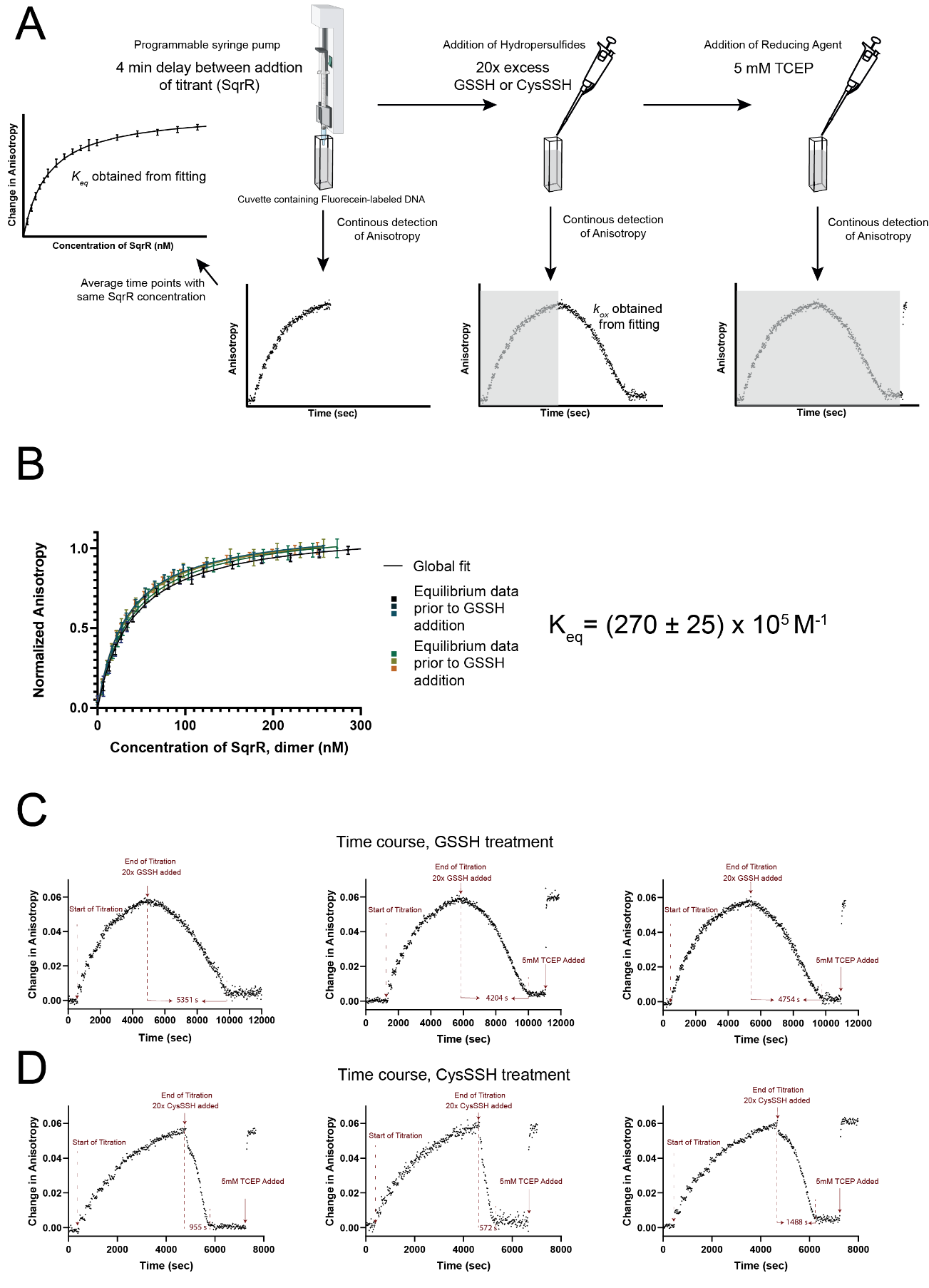


**Figure S10.** Fluorescence anisotropy experiments. (A) Schematic diagram showing the experimental procedure followed to obtain both the equilibrium association constants and the kinetic constants of the DNA dissociation process. SqrR was titrated into a quartz cuvette containing a fluorescein-labeled *rcc1451* SqrR operator using an automatic injector, and changes in fluorescence anisotropy were followed continuously over time. Each time point corresponds to a volume of titrant added to the cuvette, thus the change in anisotropy can be plotted against the SqrR concentration by averaging all the anisotropy values for a particular volume. When saturation of the operator binding sites occurred, titraton was stopped and a 20-fold excess of polysulfides with respect to SqrR concentration was added into the cuvette. The anisotropy was monitored until a new equilibrium condition was reached. These data allowed us to obtain a kinetic constant for the oxidation of SqrR with different hydropolysulfides. Finally, 5 mM TCEP was added as a reducing agent to test reversibility of SqrR oxidation. (B) Binding Isotherms for reduced SqrR titrated into *rcc1451* operator. Data was fitted to a rectangular hyperbola (number of sites = 1) for each experiment, obtaining an average binding constant (*K*_eq_) (Table 3). A total of six (6) independent experiments (later treated with GSSH depicted in *black*, *blue* and *cyan* and with CysSSH depicted in *green*, *yellow*, and *orange*). Points and error bars indicate, respectively, mean, and standard deviation of 16 points taken for each SqrR concentration in each experiment. (C) Kinetics of DNA release upon treatment with GSSH of SqrR following experimental scheme depicted in panel (A). (D) Kinetics of DNA release upon treatment with CysSSH of SqrR following experimental scheme depicted in panel (A). The three experiments performed under each condition represent independent replicates.


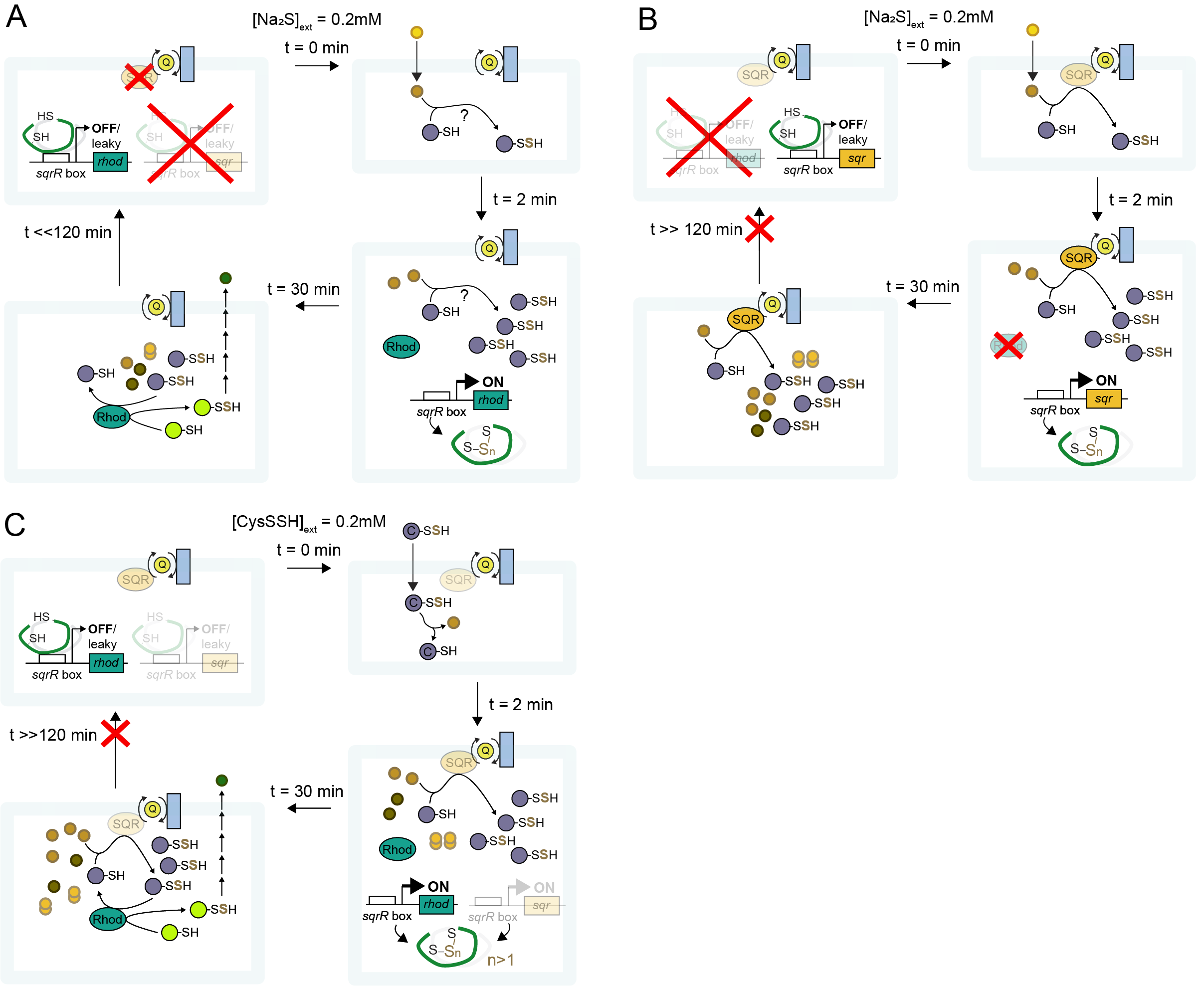


**Figure S11.** Schematic of polysulfide impact on SqrR-mediated sulfide-induced transcription for (A) Δ*sqr* cells stressed with Na_2_S, (B) Δrcc01557 (Rhodanese, sulfurtransferase) cells stressed with Na_2_S and (C) Δsqr and/or wild-type cells stressed with exogenous CysSSH.

**SI References**

1. Sato T, Inoue K, Sakurai H, Nagashima KVP (2017) Effects of the deletion of *hup* genes encoding the uptake hydrogenase on the activity of hydrogen production in the purple photosynthetic bacterium *Rubrivivax gelatinosus* IL144. *Journal of General and Applied Microbiology* 63(5):274–279.

2. Sganga MW, Bauer CE (1992) Regulatory factors controlling photosynthetic reaction center and light-harvesting gene expression in *Rhodobacter capsulatus*. *Cell* 68(5):945–954.

3. Shimizu T, Cheng Z, Matsuura K, Masuda S, Bauer CE (2015) Evidence that altered *cis* element spacing affects PpsR mediated redox control of photosynthesis gene expression in Rubrivivax gelatinosus. *PLoS ONE* 10(6):e0128446.

4. Masuda S, Bauer CE (2004) Null Mutation of HvrA Compensates for Loss of an Essential *relA*/*spoT*-Like Gene in *Rhodobacter capsulatus*. *Journal of Bacteriology* 186(1):235–239.

5. Akaike T, et al. (2017) Cysteinyl-tRNA synthetase governs cysteine polysulfidation and mitochondrial bioenergetics. *Nature Communications* 8:1177.

6. Takata T, et al. (2021) Methods in sulfide and persulfide research. *Nitric Oxide* 116(September):47–64.

7. Shimizu T, et al. (2017) SqrR functions as a master regulator of sulfide-dependent photosynthesis. *Proceedings of the National Academy of Sciences of the United States of America* 114(9):2355–2360.

8. Capdevila DA, et al. (2020) Structural basis for persulfide-sensing specificity in a transcriptional regulator. *Nature Chemical Biology*. doi:10.1038/s41589-020-00671-9.

9. Banerjee R, et al. (2015) Assay Methods for H_2_S Biogenesis and Catabolism Enzymes Ruma. *Methods Enzymol* 554:189–200.

10. Arieli B, Padan E, Shahak Y (1991) Sulfide-induced sulfide-quinone reductase activity in thylakoids of *Oscillatoria limnetica*. *Journal of Biological Chemistry* 266(1):104–111.

11. Shahak Y, Arieli B, Padan E, Hauska G (1992) Sulfide quinone reductase (SQR) activity in *Chlorobium*. *FEBS Letters* 299(2):127–130.

12. Young DA, Bauer CE, Williams JC, Marrs BL (1989) Gentic evidence for superoperonal organization of genes for photosynthesis pigments and pigment-binding proteins in *Rhodobacter capsulatus*. *Molecular & General Genetics* 218(1):1–12.

13. Kuzmič P (1996) Program DYNAFIT for the analysis of enzyme kinetic data: Application to HIV proteinase. *Analytical Biochemistry* 237(2):260–273.

14. Shen J, et al. (2018) Hydrogen Sulfide Sensing through Reactive Sulfur Species (RSS) and Nitroxyl (HNO) in *Enterococcus faecalis*. *ACS Chemical Biology* 13(6):1610–1620.

15. Fakhoury JN, Capdevila DA, Giedroc DP (2022) Protocol for using organic persulfides to measure the chemical reactivity of persulfide sensors. *STAR Protocols* 3(2):101424.

16. Zhang Y, et al. (2022) Discovery and structure of a widespread bacterial ABC transporter specific for ergothioneine. *Nature Communications* 13:7586.
